# Delayed-matching-to-position working memory in mice relies on NMDA-receptors in prefrontal pyramidal cells

**DOI:** 10.1038/s41598-021-88200-z

**Published:** 2021-04-22

**Authors:** Kasyoka Kilonzo, Bastiaan van der Veen, Jasper Teutsch, Stefanie Schulz, Sampath K. T. Kapanaiah, Birgit Liss, Dennis Kätzel

**Affiliations:** 1 Institute of Applied Physiology, Ulm University, Albert-Einstein-Allee 11, 89081 Ulm, Germany; 2 Linacre College and New College, University of Oxford, Oxford, UK; 3 Present Address: Newcastle University, Newcastle upon Tyne, UK

**Keywords:** Diseases of the nervous system, Schizophrenia, Neuroscience, Learning and memory, Cortex, Habituation, Short-term memory, Spatial memory, Working memory

## Abstract

A hypofunction of N-methyl-D-aspartate glutamate receptors (NMDARs) has been implicated in the pathogenesis of schizophrenia by clinical and rodent studies. However, to what extent NMDAR-hypofunction in distinct cell-types across the brain causes different symptoms of this disease is largely unknown. One pharmaco-resistant core symptom of schizophrenia is impaired working memory (WM). NMDARs have been suggested to mediate sustained firing in excitatory neurons of the prefrontal cortex (PFC) that might underlie WM storage. However, if NMDAR-hypofunction in prefrontal excitatory neurons may indeed entail WM impairments is unknown. We here investigated this question in mice, in which NMDARs were genetically-ablated in PFC excitatory cells. This cell type-selective NMDAR-hypofunction caused a specific deficit in a delayed-matching-to-position (DMTP) 5-choice-based operant WM task. In contrast, T-maze rewarded alternation and several psychological functions including attention, spatial short-term habituation, novelty-processing, motivation, sociability, impulsivity, and hedonic valuation remained unimpaired at the level of GluN1-hypofunction caused by our manipulation. Our data suggest that a hypofunction of NMDARs in prefrontal excitatory neurons may indeed cause WM impairments, but are possibly not accounting for most other deficits in schizophrenia.

## Introduction

Working memory (WM) is the cognitive function that allows humans and other animals to transiently hold items of thought or perception at the forefront of attention in order to guide goal-directed behaviour^1,2^. This capacity is compromised in many psychiatric disorders, including schizophrenia^3^ but also attention-deficit-hyperactivity disorder, major depression, and bipolar disorder, as well as in some neurodegenerative disorders like Parkinson’s disease and Alzheimer’s-type dementia^4,5^. WM deficits respond only poorly, if at all, to currently available medication^4^. Therefore, it is key to understand the mechanisms underlying WM in order to identify promising molecular targets for its improvement.

Functional imaging in humans has centrally implicated the dorsolateral prefrontal cortex (dlPFC) in WM^6,7^. A prominent mechanistic model holds that ongoing spiking activity of excitatory cells in the dlPFC—so-called delay-activity—is the physiological correlate of the transient encoding of information in WM in primates^8,9^. WM is assessed in tasks that demand a behavioural response to a briefly presented sensory cue, albeit only after some waiting time during which the cue is not present (*delayed response*). Some WM tasks also demand a delayed motor response that is the opposite of the prior action (*delayed alternation*). During such tasks, delay-activity is observed in single prefrontal neurons of monkeys across the time period that starts with cue presentation and ends with the behavioural response whereby many of these cells fire only in response to specific cues but not others^10–20^.

The cellular basis of this prefrontal delay-activity remains far from understood. A recent study in monkeys has determined that the expression of N-methyl-D-aspartate (NMDA) glutamate receptors (NMDARs) in neurons of the dlPFC is essential for delay-activity of its excitatory cells during a WM task^21^. The same study also demonstrated that *systemic* pharmacological inhibition of NMDARs by ketamine impairs WM performance^21^. NMDA-receptors are a convergence point of multiple other key modulators of prefrontal delay-activity including nicotinic acetylcholine receptors^22^ or $\mathrm{\alpha }$1- and $\mathrm{\alpha }$2-adrenoreceptors^23^. Further, the particularly slow inactivation time constant of NMDARs containing the GluN2B subunit make them particularly suitable to sustain excitation of prefrontal neurons over time^24^. Accordingly, genetic ablation of GluN2B across neocortical and hippocampal excitatory cells in mice impaired T-maze alternation performance, a rodent spatial working memory (SWM) assay—but also other forms of memory involving spatial processing in general^25^.

However, direct evidence, that NMDARs in prefrontal excitatory neurons are essential for working memory (not only delay-activity) is still lacking. This knowledge is essential to close the logic of the argument that NMDAR-mediated delay-activity in prefrontal pyramidal cells underlies the maintenance of the information during the delay-phase. It also has wider implications for understanding the severe WM impairments in schizophrenia that have been associated with pathological signal processing in the prefrontal cortex^26,27^. Independently, it has been hypothesized that this disorder is caused by a hypofunction of NMDARs^28–30^. The mechanistic link between NMDAR-hypofunction and WM impairments in schizophrenia have, however, remained unclear.

Therefore, we here investigated whether NMDARs in prefrontal excitatory neurons are necessary for SWM performance in mice. We focused on the rodent homologue of the dlPFC—the medial prefrontal cortex^31,32^, especially its prelimbic subdivision (PrL)—which has been implicated in SWM before^33–35^. However, we deployed a recently developed murine delayed-matching-to-position (DMTP) operant task that emulates the rat *combined attention and memory* (CAM) task^36–38^ and uses a 5-choice wall to assess SWM (5-CSWM)^39^. In contrast to the standard rodent WM assay of alternation in the T-maze, the 5-CSWM task lacks potential confound by novelty-preference, reduces mediating strategies that are possible in most other operant SWM tasks^33^, and enables control over core variables like stimulus-specific attention and motivation^39^. Additionally, we also applied the T-maze alternation task for comparison and assessed a wide range of other behavioural functions—particularly those relevant to schizophrenia, like sustained attention, spatial short-term habituation, novelty-induced locomotion, anhedonia, and social functioning.

## Results

### Ablation of NMDA-receptors in prefrontal pyramidal cells

**Figure 1:**
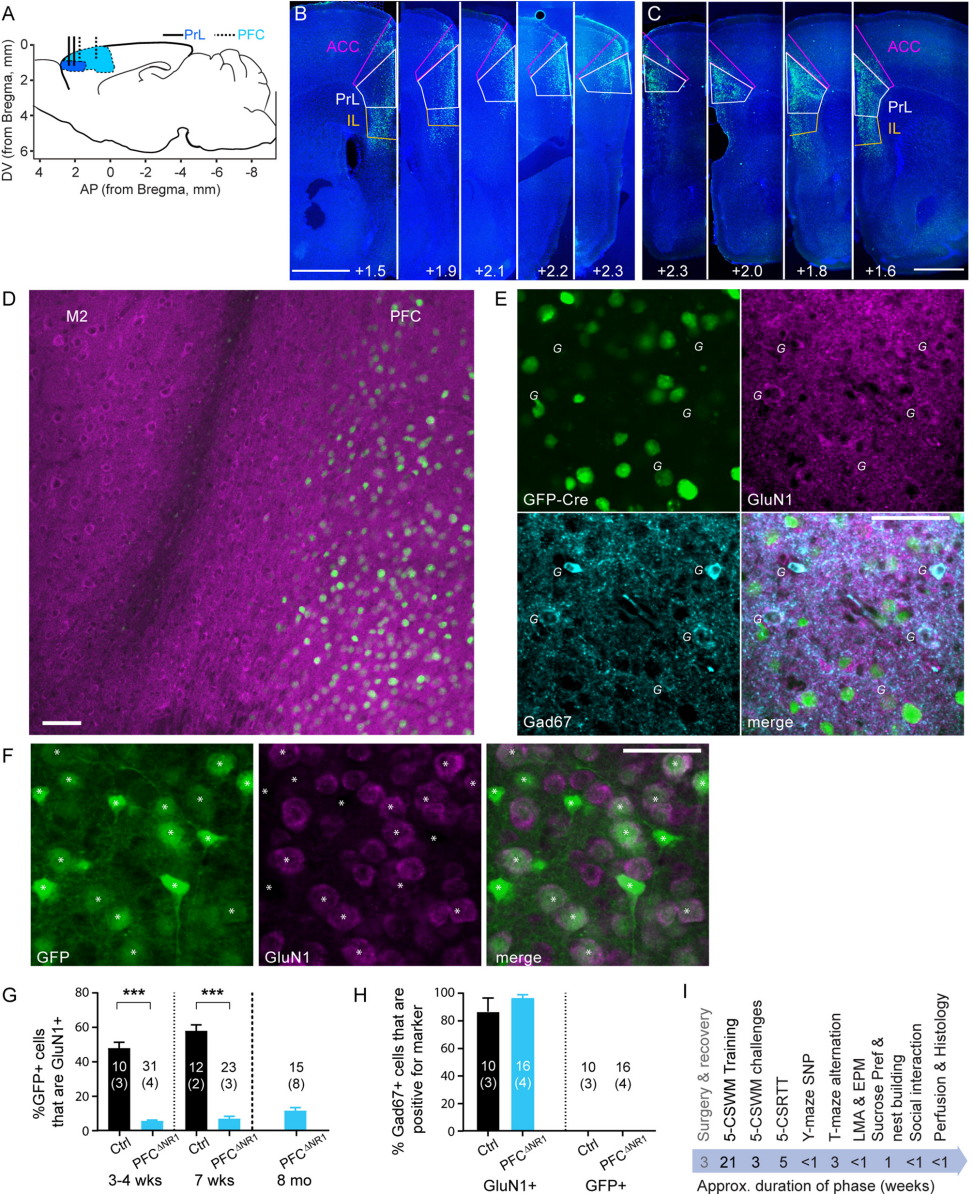
Virally mediated NMDAR ablation. (**A**) Illustration of target region of the medial PFC (blue), with the PrL region in darker shade of blue, in a saggital cut through the mouse brain. Vertical lines indicate infusion sites for broader (dotted, dorsal PFC) and more narrow (solid, PrL) targeting. (**B, C**) Examples of staggered coronal slices of a mouse brain transfected with rAAV8-CamKII$\mathrm{\alpha }$-GFP-Cre (green) either with broader (B) or more narrow (C) targeting (see Methods). Blue, DAPI-stain; green, native GFP-fluorescence. Scale bar, 1 mm. Numbers state the distance from Bregma (as shown in A). (**D**) Microscopic image showing expression of GFP (green) and GluN1 (magenta) of a brain slice (at the PFC/M2 border) of an fGrin1 mouse brain slice transfected with a GFP-Cre vector; note the ample expression of GluN1-positive cells outside the transfected region. (**E**) Closer view onto a section of GFP-Cre-transfected (green) PFC double-stained against GluN1 (magenta) and Gad67 (cyan). Italic *G* are placed to the bottom left of Gad67-positive cells. (**F**) Section of a PFC of a GFP-transfected control mouse, stained against GluN1. Note that GFP-expression from the control vector is cytosolic (F), but GFP is localized to the nucleus if fused to Cre (E). GFP-positive cells are labelled by white asterisks. Scale bar, 50 $\upmu$m (D-F). (**G**) Bar-graphs showing average share of GFP-positive cells that were also positive for GluN1 in the indicated groups and at the stated time-points after transfection. (**H**) Average share of Gad67-positive cells that were also positive for GluN1 (left) or GFP (right) in the indicated groups (the time points 3–4 wks and 7 wks after transfection were merged for this analysis; no Gad67-positive cells were GFP-positive). Stated *N*-numbers in each bar of (G-H) refer to analysed fields of view (top) obtained from the number of mice stated in brackets below. ***P < 0.001, MWU-test. (**I**) Order and approximate duration of behavioural assays (black) conducted in this study after the initial surgery (grey).

We ablated NMDA-receptors specifically from PFC excitatory cells by injection of an rAAV-vector containing an expression cassette in which the CamKII$\mathrm{\alpha }$-promoter drove expression of the *Cre*-recombinase gene fused to *GFP* (GFP-Cre) into 28 male C57BL/6N mice in which the *Grin1* gene encoding the obligatory NMDAR subunit GluN1 (NR1) was floxed^40^ (fGrin1 mice; PFC^ΔNR1^ group), as done previously^41,42^. In these infusions, the dorsal PFC comprising the PrL and the anterior cingulate cortex (ACC) was targeted, although expression was often also found in the infralimbic (IL) division immediately ventral to the PrL (Fig. 1A–C). Eight animals that had no or only unilateral expression in the PrL were excluded from all subsequent analysis *post-hoc* (Supplementary Table 1). The control group (Ctrl) comprised 13 male fGrin1 littermates that received a similar rAAV-vector encoding only GFP in the expression cassette.

To confirm NMDAR ablation, we immuno-stained coronal brain sections from separate mice that were perfused 3–4 or 7 weeks after viral transfection with anti-GluN1 antibodies. Among GFP-Cre-expressing cells, the proportion with detectable NMDAR immuno-signal was strongly reduced compared to cells expressing only GFP at both time points (Fig. 1D–G). Notably, however, GluN1 was still detectable in a small (< 10%) fraction of GFP-Cre-positive cells, and it was *not* detected in 40–50% of GFP-positive cells in the control group (Fig. 1G). The combination of these observations suggests that the difference between the two groups is a matter of degree rather than a binary difference. We did the same analysis on slices from PFC^ΔNR1^ mice of the behavioural cohort and found a similarly low level of GluN1-expression (Fig. 1G). We also confirmed that GFP-Cre expression did not occur in Gad67-positive cells, i.e. inhibitory interneurons, and that GluN1 expression in these neurons displayed no difference between the Cre-transfected and the control group (Fig. 1E,H).

### NMDAR ablation from prefrontal excitatory cells does not impair primary acquisition of the 5-CSWM task

After recovery from surgery, fGrin1 mice were taken through a series of behavioural tests (Fig. 1I) which started with the 5-CSWM task assessing DMTP WM that required a long training period (11–21 weeks before testing started; Supplementary Fig. 1). This assay consisted of a sample phase (SP) in which mice needed to poke into an illuminated hole in the 5-choice wall, a delay phase (DP) during which they had to transition to the illuminated reward receptacle at the opposite wall of the operant chamber, and a choice phase (CP) where a second hole was illuminated alongside the originally poked hole and mice were rewarded for choosing the latter, realizing a DMTP rule^39^ (Supplementary Fig. 1; see Methods for details). The mice acquired this task through multiple, incrementally more difficult training stages (Supplementary Fig. 1) until reaching a final baseline stage (stage 4) in which the duration of the stimulus presentation (SD) in the SP was shortened to 8 s while the DP duration was still 2 s posing relatively little WM demand.

**Figure 2:**
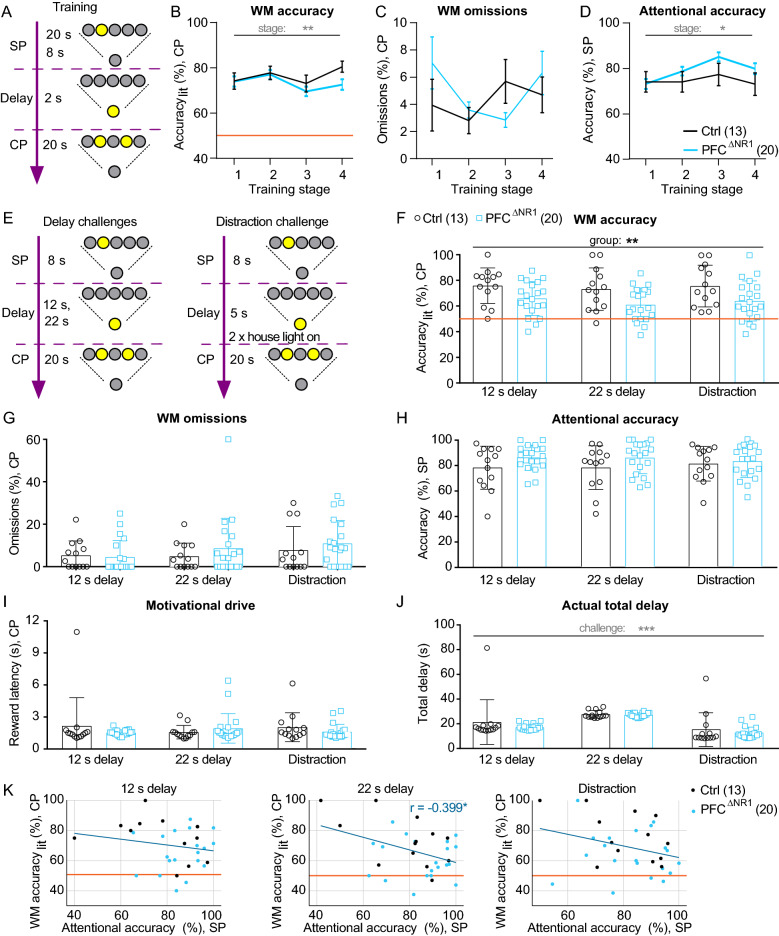
Training and behavioural challenges in the DMTP 5-CSWM task. (**A**) Simplified scheme of the 5-CSWM operant DMTP assay as conducted during the training stages 1–4 (see Supplementary Fig. 1 for details). (**B-D**) Average performance on the last 3 d of each of the four training stages for the primary WM indicator *accuracy*_*lit*_ measured in the CP (B), an indicator of task engagement, *% omissions* in the CP (C), and the primary attention indicator *accuracy* measured in the SP (D). See Supplementary Fig. 1 for details on stage parameters, further performance indicators for these sessions and the averages of the *first* 3 sessions on each stage, and for statistical details on RM-ANOVAs for all assessed parameters. (**E**) Illustration of the applied delay and distraction challenges. (**F–J**) Average performance and control parameters across the three challenge conditions (data from the first challenge day each conducted immediately after a day with training in the baseline stage) showing the primary WM indicator CP *accuracy*_*lit*_ (F), *% omissions* in the CP (G), attentional SP *accuracy* (H), CP reward latency as a metric for motivational drive (I), and the true total delay (J). See Supplementary Fig. 2 for further performance indicators. Statistical indicators in every data panel relate to RM-ANOVA across indicated protocols and groups, stating effects of protocol (grey) or group (black); no significant group-protocol interactions were found. (**K**) Scatter plots of WM *accuracy*_*lit*_ (CP) and attentional accuracy in the corresponding SP for all three WM challenges (named above each sub-panel); individual dots represent animals, colour-coded by group; dark blue line represents linear fit across all animals. A significant negative correlation (r) was found in the 22 s delay challenge (middle), if including all animals—but not for the KO-group alone. See Supplementary Table 2 for statistical details on RM-ANOVAs for all assessed parameters, and Supplementary Table 3 for additional ANCOVAs and correlation analysis (relating to K) exploring dependencies between WM accuracy and attentional accuracy. The legends in (D) and (F) indicate the colour-code and size of each group. Orange lines (B, F) represent chance-level performance. *P < 0.05, **P < 0.01, ***P < 0.001. Error bars, s.e.m. (B–D) or S.D. (F–J).

We analysed training progress by averaging performance indicators during the first and last 3 training sessions conducted on each of the 4 main training stages. WM performance was assessed by determining the relative amount of correct CP choices normalized to the sum of CP choices of the correct and the incorrect illuminated holes (WM *accuracy*_*lit*_; 50% being chance level performance). The relative amount of omitted CP responses (%omissions) served as a measure for WM task engagement. Attentional performance was measured in the *SP* as *accuracy*, i.e. the number of correct SP responses normalized to the total number of SP responses, analogously to the accuracy measure in the 5-choice-serial-reaction time task (5-CSRTT)^43^. Training progress was indicated by a significant improvement of all three parameters across beginnings of the four training stages (*P* < 0.00005; effect of stage, repeated-measures (RM) ANOVA; Supplementary Fig. 1) and the improvement of the accuracies across the last days of the training stages (*P* < 0.005; effect of stage; Fig. 2A–D; see Supplementary Table 2 for statistical details on this and further 5-CSWM analysis). In contrast, there were no significant effects of group for any performance parameter of this task—including secondary indicators of training progress and motivation like reward latency and total number of obtained SP and CP rewards—in the overall ANOVA (Fig. 2B–D; Supplementary Fig. 1). There was, however, a trend for a group-stage interaction in the secondary WM indicator accuracy_all_ (number of correct responses divided by number of all active responses), driven by lower WM performance in the PFC^ΔNR1^ mice in the last stage (*P* = 0.030, Sidak post-hoc test, Supplementary Fig. 1). We investigated this further by comparing performance between the two groups on individual days of the final stage, and found that PFC^ΔNR1^ mice trended to display lower WM accuracy on days after a training gap but not on days that followed a previous training on the same stage. This qualitative observation suggests that our prefrontal NMDAR-ablation does not impair the acquisition of the task, but nevertheless entails some difficulty to maintain high performance.

### NMDAR ablation from prefrontal excitatory cells impairs DMTP working memory when challenged

Once training was completed, three challenge protocols were conducted to interfere with memory maintenance and thereby test WM performance. Firstly, the delay-time across which the memory needed to be actively maintained was increased from the 2 s baseline to 12 or 22 s (*delay-challenge*); secondly, the animals were distracted by two randomly timed switches of the house-light (for 0.5 s each) during a moderately increased delay of 5 s (Fig. 2E). Challenges were conducted on two consecutive days per protocol and animals received training at baseline stages for 2 d during those challenge blocks. A RM-ANOVA across groups and those three challenge conditions revealed a significant effect of group on WM performance (*accuracy*_*lit*_) (*P* = 0.0025, assessing performance on the first challenge days only; *P* = 0.0066, assessing performance as the average of both days of each challenge). This effect was driven by a reduced accuracy in PFC^ΔNR1^ mice, with no effect of challenge or interaction (*P* > 0.4; Fig. 2F). The same pattern was seen for the secondary WM indicator accuracy_all_, while no other indicator assessed on this task showed a significant effect of group or a group-challenge interaction (Fig. 2G–J; Supplementary Fig. 2; Supplementary Table 2). Importantly, groups did not differ with respect to indicators of task engagement (CP and SP omissions rates), motivation (reward latency, number of consumed rewards), attentional (SP) accuracy, response latency, the true delay time between SP and CP response (i.e. the sum of CP-initiation latency, set delay, and CP-response latency), or the number of correct and incorrect CP premature responses, which could indicate a form of mediation (Fig. 2G-J; Supplementary Fig. 2; Supplementary Table 2). This pattern of results suggested that, across challenges, mice with selective ablation of NMDARs in excitatory cells of the medial PFC showed a specific deficit in DMTP WM performance.

To scrutinize this conclusion further we firstly included the three most likely alternative mediators of this group difference as covariates of RM-ANOVAs evaluating WM-group associations: *sustained attention* as assessed from either the SP attentional accuracy or the baseline or attention challenge of the subsequently conducted 5-CSRTT (see below), *SP omissions*, and the *true total delay*. In the latter two cases, the effect size of group on WM accuracy (10.8 without covariates) either increased (SP omissions, *F* = 16.0) or remained in the same range (*F* = 9.9, true delay; Supplementary Table 3), indicating that WM-differences between groups were independent from those two factors. There was also no correlation between the true delay and WM performance (-0.1 < *r* < 0.3; *P* > 0.3; bivariate Pearson correlations within each genotype and challenge condition)*.* However, when including any of the indicators of attentional accuracy, the effect size of group decreased somewhat (*F* = 6.3 with inclusion of SP accuracies; *F* = 7.4 with inclusion of 5-CSRTT accuracies; Supplementary Table 3). This indicates that this factor may be involved in mediating differences in WM performance. To explore this further, we calculated bivariate Pearson correlations between parameters of attentional accuracy and WM accuracy in each of the three challenge conditions (Supplementary Fig. 3; Fig. 2K). WM performance correlated significantly and inversely with SP accuracy in the 22 s delay challenge if including *all* mice (Fig. 2K). However, no such correlation was found in the other two challenges nor in the same challenge if including only PFC^ΔNR1^ mice (*r* = -0.06; Fig. 2K; Supplementary Table 3). These rather inconsistent correlations, their inverse nature where present, and the remaining significant effects of group on WM accuracy after inclusion of such covariates (Supplementary Table 3), suggest that attentional differences did not mediate WM differences associated with prefrontal NMDAR ablation, but rather exerted a minor influence in the control group.

We further explored the possibility that the lower performance in knockouts was caused by the unexpected switch of the task protocol rather than by a genuine WM impairment, given the variation of performance observed before with training breaks in the baseline stage (Supplementary Fig. 1M). As each challenge was conducted on two consecutive days, we repeated the RM-ANOVA analysis for the second day alone. In this case the effect of group reached only trend level (*F* = 2.91; *P* = 0.098). However, the inspection of the individual data on both days of each challenge showed that there was no significant improvement of performance on the second day compared to the first day in PFC^ΔNR1^ mice (*P* > 0.3, paired *t*-tests; Supplementary Fig. 3). Nevertheless, marginal increases of the mean performance of PFC^ΔNR1^ mice on the second days compared to the first days of each challenge—in combination with the reverse effect in the control group and an overall higher variability across mice on the second days—were likely a main driver of the loss of a significant effect of group on the second day (Supplementary Fig. 3).

Further, given the high SP omission rates (Supplementary Fig. 2), we assessed if mice would generally use mediation to solve this task by omitting to respond in one half of the box (encoding correct choices by body-positioning). To assess this, we analysed data from a separate group of 11 wildtype mice that had been trained in the same task in a custom-designed operant box system, that allowed us to record every individual event and action of the animal. Each animal used every hole to respond in the SP and CP of this task, usually not showing a strong side-bias (Supplementary Fig. 3), which suggests that, at least unimpaired mice, do not use a strategy of selective omission.

### NMDAR ablation from prefrontal excitatory cells does not impair T-maze rewarded alternation

**Figure 3:**
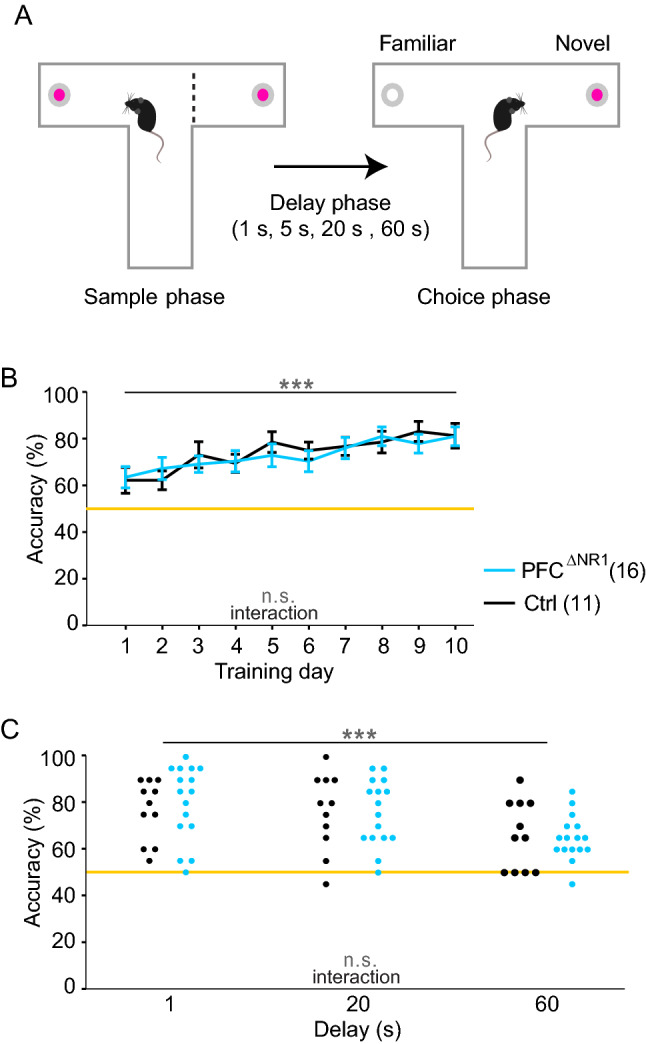
T-maze rewarded alternation assay of DNMTP WM. (**A**) Illustration of T-maze task, in which each trial consists of a rewarded sample phase and a rewarded choice phase, separated by a delay of 5 s during training, or 1 s, 20 s or 60 s during challenges. (**B**) Average alternation performance across the 10 trials of a session over the 10 training days. (**C**) Performance of individual mice (dots, colour-coded as in (B)) in three delay challenges, as indicated. Grey statistical indicators relate to RM-ANOVA, with asterisks on horizontal lines indicating effect of training (B) or delay (C); no significant effect of group was found. The legend indicates the colour-code and size of each group. Orange line, chance level performance. n.s., *P* > 0.1, ***P < 0.001. Error bars, s.e.m.

We further determined, if this WM deficit was also detectable by the most commonly used rodent assay of delayed-*non*-matching-to-position (DNMTP) SWM, namely T-maze rewarded alternation^44^ (Fig. 3A). Mice were first trained over 10 sessions with 10 trials each featuring a delay of 5 s. Across these sessions and groups, alternation performance improved significantly (*P* < 0.0001; RM-ANOVA), but no effect of group or interaction could be detected (*P* > 0.1; RM-ANOVA, see Supplementary Table 4 for statistical details on this and all further behavioural assays; Fig. 3B). Subsequently, WM was challenged by using, firstly, a very short (1 s) delay, and secondly longer delays (20 s and 60 s)^45^. Each protocol was applied over 2 consecutive days (10 trials/d) and the results were averaged across those 2 days. In an RM-ANOVA across the three challenges, there was a significant effect of delay (*P* = 0.001), driven mainly by lower performance with 60 s delay compared to the two other paradigms (Fig. 3C). However, no significant effect of group or group-delay interaction was detectable (*P* > 0.1) across challenges (RM-ANOVA), not even when analysing each condition separately (*t*-test; Fig. 3C). These results suggest that T-maze alternation performance is not dependent on NMDARs in prefrontal excitatory cells.

### Ablation of NMDARs from PFC excitatory cells does not impair sustained attention

**Figure 4:**
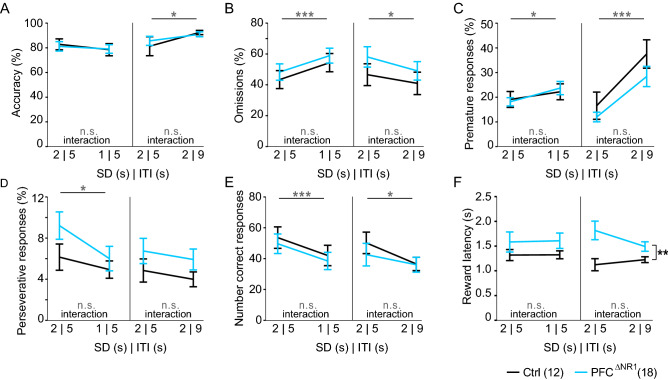
5-choice-serial-reaction-time task (5-CSRTT) performance. Results of attention challenge (reduction of stimulus duration, SD, from 2 to 1 s; left) and impulsivity challenge (increase of inter-trial-interval, ITI, from 5 to 9 s; right) across six key parameters of the 5-CSRTT. (**A**) Accuracy as primary measure of attention, (**B**) relative omissions as a measure of task engagement and attentiveness, (**C**) relative premature responses as primary measure of waiting impulsivity, (**D**) relative perseverative responses, (**E**) total number of correct responses (earned rewards), and (**F**) latency to collect the reward as a compound proxy for locomotor drive and motivation. Grey statistical indicators relate to RM-ANOVA, with asterisks on horizontal lines indicating effect of challenge, and asterisks on vertical lines referring to an effect of group; no significant challenge-group interactions were found. The legend indicates the colour-code and size of each group. See Supplementary Table 4 for all related statistics. n.s., *P* > 0.1, *P < 0.05, **P < 0.01 ***P < 0.001. Error bars, s.e.m.

As the 5-CSWM data suggested a possible mild improvement of sustained attention in PFC^ΔNR1^ mice, we further trained and tested the cohort on the 5-CSRTT, i.e. a reduced version of the 5-CSWM task they had previously performed, containing only the SP. Mice were trained on a standard 5-CSRTT protocol with a 5 s inter-trial-interval (ITI) and first a 4 s SD, and then a 2 s SD (baseline). Subsequently, the cohort was exposed to a challenge of attention (reduction of SD to 1 s) and, separately, to a challenge of impulsivity (increase of ITI from 5 to 9 s) for one session per challenge. Irrespective of protocol (baseline or challenges), we did not detect any differences between the groups on measures related to attention (accuracy, omissions; Fig. 4A,B), impulsivity (premature responses; Fig. 4C) or perseveration (perseverative responses; Fig. 4D) under any of the baseline and challenge conditions (*P* > 0.1, RM-ANOVAs across groups and each baseline-challenge pair; Supplementary Table 4). The challenges, however, were successful in increasing impulsivity (premature responses, both challenges, Fig. 4D) and omission rates (attention challenge only, Fig. 4B; thereby also decreasing the absolute number of correct responses, Fig. 4E), as indicated by significant effects of challenge (*P* < 0.05, RM-ANOVAs, Supplementary Table 4). Across all analyzed variables, only the reward latency showed a significant effect of group in the impulsivity challenge (*P* = 0.004, RM-ANOVA), which was driven by a higher reward latency in the PFC^ΔNR1^ group compared to the control group in the impulsivity challenge and its baseline (*P* < 0.05, Sidak; Fig. 4F). However, this group-difference did not reach significance in the attention challenge and its baseline (*P* = 0.154), and was not observed in the prior 5-CSWM task (Supplementary Table 2); so it is difficult to conclude a genuine link between prefrontal NMDAR-hypofunction and motivational drive from this data. Altogether, these results suggest that NMDARs in prefrontal excitatory cells are likely not essential for sustained attention, impulsivity, or perseveration.

### Ablation of NMDARs from prefrontal excitatory cells does not impair spatial short-term habituation

**Figure 5:**
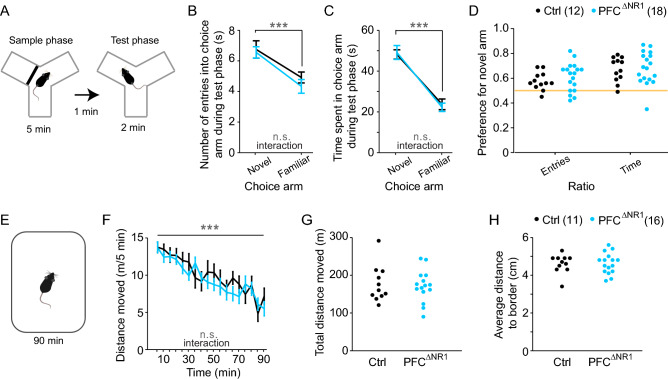
Assessment of spatial short-term habituation. (**A**) Illustration of the spatial novelty-preference test which consists of a 5 min sample phase, a 1 min ITI, and a 2 min test phase. (**B, C**) Number of entries (B) and time spent (C) in the novel and familiar choice arms during the test phase. Grey indicators, effect of arm and group-arm interaction in overall RM-ANOVA. (**D**) Preference ratios for the novel arm calculated based on the data in (B) and (C). (**E**) Illustration of novelty-induced hyperlocomotion test. (**F**) Locomotor activity measured in moved distance in 5 min intervals over the 90 min of the test. Grey indicators, effect of time bin and group-time interaction in overall RM-ANOVA. (**G**) Total locomotor activity over 90 min of individual mice (dots, colour-coded as in (F)). (**H**) Same display as in (G) but for the average distance to the border of the open field over 90 min as a proxy for anxiety. No effects of group were found (RM-ANOVA, B, C, F; *t*-test, D, G, H). The legend indicates the colour-code and size of each group. See also Supplementary Table 4 for all related statistics. n.s., *P* > 0.1, ***P < 0.001. Error bars, s.e.m.

Given that the T-maze alternation task could theoretically be solved by spatial novelty-preference (SNP), a rather passive form of short-term memory that relies on short-term habituation^46^, we assessed this parameter using the Y-maze SNP test^47^. In this task, mice explore the start arm and one of the goal arms during a 5 min SP in a Y-maze with transparent walls and—after a delay of 1 min—they are returned to the maze for a 2 min test phase during which they can access the second (novel) goal arm as well as the previously visited arms (Fig. 5A). During the test phase, all groups showed a significantly higher exploration of the novel goal arm compared to the familiar goal arm (*P* < 0.0001 for the difference between the novel and the familiar goal arm in numbers of entries and the time spent in choice arm, RM-ANOVA). However, there were no significant effects of group or group-arm interactions (*P* > 0.1, RM-ANOVA, Fig. 5B,C), and no group-differences in the preference for the novel over the familiar arm (*P* > 0.1, *t*-test Fig. 5D).

We also assessed spatial short-term habituation by measuring novelty-induced hyperlocomotion and its adaptation over a 90 min period in an open field (Fig. 5E). Across groups, a significant decrease of distance moved was seen over time (*P* < 0.0001, RM-ANOVA over 5 min intervals, Fig. 5F), but no effect of group or interaction were apparent (*P* > 0.1, RM-ANOVA). These data suggest that spatial short-term habituation is not affected by NMDAR-ablation in prefrontal excitatory cells. Also, there were no differences between the groups in total distance moved or in the average distance to the border of the open-field, an indicator of anxiety (*P* > 0.1, *t*-test; Fig. 5G,H, Supplementary Table 4). These findings of unaltered novelty preference might offer an explanation, why we only detected a WM deficit in PFC^ΔNR1^ mice in the DMTP test (Fig. 2) but not in the T-maze (Fig. 3)^46^.

### Ablation of NMDARs from prefrontal excitatory cells does not impair affective functions

**Figure 6:**
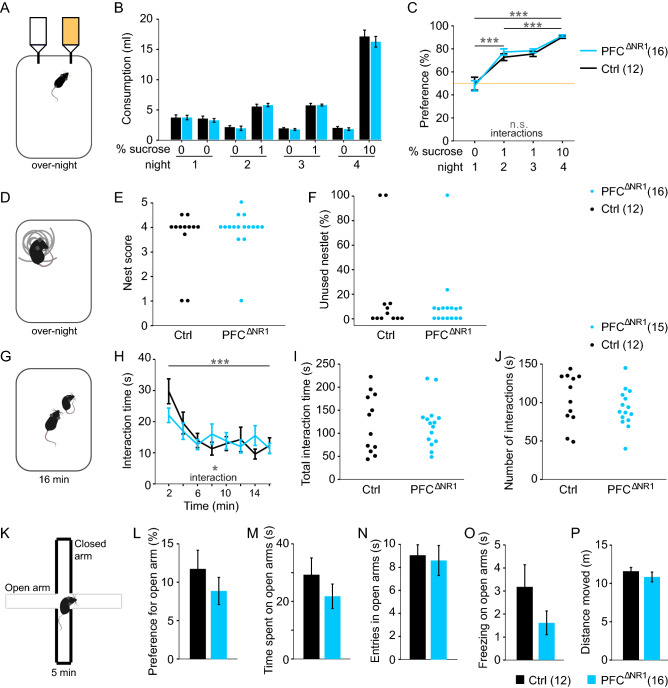
Assessment of social and emotional functioning**.** (**A**) Illustration of sucrose preference test conducted over-night while animals are single-housed. (**B**) Consumption of drinking water from each of the two bottles containing the stated concentration of sucrose across 4 nights. (**C**) Same data as in (B) but expressed as sucrose preference ratio (100*volume of water consumed from the designated sucrose-bottle/total water consumption) in %. Orange line shows chance level. Statistical indicators refer to RM ANOVA across the indicated nights and the two groups; there were no significant effects of groups or interactions. (**D**) Illustration of the nest building test. (**E, F**) Dot-graphs displaying the nest score and relative amount of unused nestlet (intact bits of nestlet heavier than 0.1 g) for each mouse. Analysis with MWU-test revealed no significant effect of group. (**G**) Assay of reciprocal social interaction in a novel open field. (**H**) Time spent in active social interactions in 2 min intervals over the 16 min test. Grey indicators, effect of time bin and group-time interaction in overall RM-ANOVA; no effect of group was found. (**I, J**) Dot-graphs displaying the total time (I) and number (J) of interactions made during the complete test; no effect of group was found (*t*-test). (**K**) Illustration of the elevated plus-maze with two open arms (grey) and two closed arms (black). (**L**) Preference to spent time on the open arm relative to the time in closed arms. (**M**) Total time spent on open arms, during the 5 min test. (**N**) Total entries into any of the open arms. (**O**) Total time spent freezing on the open arm. (**P**) Total distance moved during the test. No effect of group was found (*t*-test). The legends indicate the colour-code and size of each group for every experiment. See also Supplementary Table 4 for all related statistics. n.s., *P* > 0.1, *P < 0.05, ***P < 0.001. Error bars, s.e.m.

PFC dysfunction has also been associated with rodent correlates of negative symptoms of schizophrenia such as anhedonia and impaired social functioning^48,49^. We therefore assessed these functions at the end of the test battery. To measure anhedonia, mice were single-housed and subjected to a sucrose preference test across a schedule of four nights (Fig. 6A). On the first night, mice were habituated to being single-housed in cages with two drinking bottles both containing water. On the subsequent nights, one of the bottles contained water with either 1% (nights 2 and 3) or 10% sucrose (night 4). Analysis was conducted with RM-ANOVAs across all 4 nights, across nights 1 and 2 (transition to sucrose exposure) and across nights 2–4 (sucrose-only). In all cases there was a significant effect of session driven by changes in sucrose concentration (*P* < 0.0001) but no effects of group or group-session interactions (*P* > 0.1; Fig. 6B,C).

Following sucrose-preference testing, the capability of nest building was assessed on night 5 by adding a pressed cotton pad (2.3–2.6 g) into the cage that otherwise lacked additional environmental enrichment. The quality of the nest and the amount of unused nestlet were determined on the next morning^50^, yielding no difference between groups (*P* > 0.1, MWU test; Fig. 6D-F).

Subsequently, reciprocal social interaction with an unfamiliar adult stimulus mouse of the same sex and strain was assessed in an open field by manually scoring non-aggressive physical encounters and sniffing over 16 min in 2 min intervals (Fig. 6G,H). An RM-ANOVA across groups and time-bins revealed a significant effect of time (*P* < 0.0001)—indicating the expected social short-term habituation. Even though we found a significant group-time interaction (*P* = 0.032, RM-ANOVA), there was no significant difference between groups at any individual interval and also no effect of group across time (*P* > 0.05; Fig. 6H). Likewise, the total time and amount of interactions did not differ between groups (*P* > 0.1, *t*-tests; Fig. 6I,J), so that a genuine deficit in social interaction cannot be concluded.

Lastly, we investigated whether the groups differed in unconditioned anxiety using the elevated plus-maze test, but could not detect any group-related differences in the anxiety- or exploration-related measures in this task (*P* > 0.1, *t*-tests; Fig. 6K-P). In summary, NMDARs in prefrontal excitatory cells did not appear to be relevant to affective functioning, including deficits in the negative domain of schizophrenia or anxiety (Supplementary Table 4).

## Discussion

In the present study we have shown that NMDA receptors in pyramidal cells of the medial PFC are necessary for intact DMTP working memory in mice when probed in memory-demanding behavioural challenges. As observed in schizophrenia patients^51^, this WM deficit did not display an appreciable delay-dependency. These findings further support the hypothesis that NMDAR-mediated delay-activity in prefrontal excitatory neurons is necessary for the maintenance of WM contents over time^8,21^. Since other cognitive and affective functioning were broadly intact after prefrontal NMDAR-ablation, this WM deficit appeared to be highly specific and, hence, not the result of more basic impairments of attention, motivation, instrumental learning, impulse control, or preference for sweet rewards.

### Possible confounds of the DMTP working memory phenotype in PFC^ΔNR1^ mice

Even though we found a significantly lower DMTP WM accuracy in PFC^ΔNR1^ mice compared to controls—no matter if using data from the first days or both days of each challenge—performance in this group was not at chance level. We therefore explored, if this result could be due to confounds rather than a genuine WM impairment. One possible confound could be *mediation strategies*: either (a) the NMDAR-ablation could impair the capacity to deploy a mediation strategy that control animals use to improve their performance, or (b) PFC^ΔNR1^ mice use mediation to remain well above chance performance despite being impaired in genuine WM. The 5-CSWM task^39^ and its rat version^37^ were originally developed to reduce the possibility of mediation by strategies like encoding of correct choices by body-positioning that affected earlier operant assays of WM. This reduction is mainly due to the requirement to move to the opposite wall to obtain a reward, extended delays in total darkness (possibly interrupted by light-distraction), and the large amount of stimulus configurations that the animal experiences in the CP. The fact, that both rats^37,38^ and mice need several months to acquire this task—as opposed to 1–2 weeks in simpler 2-choice operant tasks^52^ or multi-choice tasks without requirement to return to the opposite wall^53^—suggests that the opportunity for mediation is indeed limited. One possibility would be that mice remain in one half of the box, omitting about half of the responses in the SP (which is indeed the omission rate in our study, Supplementary Fig. 2). An analysis of data in a separate cohort however suggested that this is not a strategy employed by wildtype mice (Supplementary Fig. 3), rendering option (a) rather unlikely. Even though we cannot currently exclude option (b)—the possibility that knockouts are impaired in WM but use mediation to perform above chance level—this scenario does not contradict our conclusion regarding the induced WM impairment. Also, in our fGrin1 cohort, SP omission levels proved to be independent from WM accuracy. Further, premature responding—a possible indicator of waiting in front of the correct hole to overcome the extended delay^39^—was not elevated in PFC^ΔNR1^ mice (Supplementary Fig. 2).

A second option would be that the difference in WM performance is caused by knockout-induced changes to another cognitive or emotional function. The broad absence of group-differences across other parameters assessed in the 5-CSWM and other cognitive and affective behavioural assays (Fig. 2, 3, 4, 5 and 6; Supplementary Fig. 2) renders this scenario rather unlikely. Nevertheless, we found that attentional accuracy—as measured in the 5-CSWM SP or the 5-CSRTT—co-varied partially with WM performance if analysed across all mice of the cohort. However, this co-variation was insufficient to account for the reduced WM performance in PFC^ΔNR1^ mice, as their lower WM accuracy remained significant after accounting for this factor and no correlation with found within the KO group alone. A further option could be that prefrontal NMDAR-knockout rather reduces the capacity to adapt flexibly to new cognitive demands, thereby causing reduced CP accuracy simply because of the introduction of the unexpected behavioural challenge. While we did not find a significant increase of WM performance of PFC^ΔNR1^ mice on the second challenge day compared to the first day in any of the protocols, there was still a *qualitative* increase (Supplementary Fig. 3), and the effect of group on WM accuracy reached only trend-level if analysing WM accuracy exclusively on the second day of each challenge. We also found reduced WM performance of PFC^ΔNR1^ mice on the baseline protocol after training breaks (Supplementary Fig. 2). Therefore, based on the present data (lacking further repetitions of the same challenge protocol), we cannot rule out that NMDAR-knockout from prefrontal excitatory cells partially impairs retention of the task rule or the cognitive flexibility needed to perform under unexpected challenge conditions, rather than WM per se. Notably, the delay challenges in the T-maze and the various challenges in the 5-CSRTT also pose abrupt changes to cognitive demands, but did not induce altered performance in the PFC^ΔNR1^ group. However, the extensive training on the 5-CSWM compared to all other tasks (Fig. 1I) might render mice more susceptible to abrupt protocol changes on the 5-CSWM and thereby reveal deficits of cognitive flexibility more easily. In summary, while a genuine impairment of DMTP WM is the most likely explanation for the behaviour of PFC^ΔNR1^ mice in the 5-CSWM task, the alternative explanation of our data as reflecting reduced cognitive flexibility remains a possibility.

### Working memory performance in the 5-CSWM and the T-maze tasks are dissociable by prefrontal NMDAR-knockout

Performance in maze-based alternation assays is impaired by ablation of GluN2B-containing^25^ or all^54^ NMDARs from excitatory neurons across the *whole* forebrain. However, blockade or knockout of NMDARs in the hippocampus alone is sufficient to impair rewarded spatial alternation^25,30,40,55,56^, in line with a key role of the hippocampus for alternation performance^57^. Knockout of NMDARs from excitatory cells in superficial layers of prefrontal and sensory neocortex, in turn, did not affect spatial alternation^58^. This left the role of NMDARs in *prefrontal* excitatory cells for rodent WM elusive.

Our data from the T-maze confirms that NMDARs in prefrontal excitatory cells are likely not necessary for spatial alternation performance, despite multiple experiments demonstrating the encoding of task-related information in the PFC^59–61^. The divergence between the results from the T-maze and the 5-CSWM task may be due to a difference in the neurophysiological underpinnings of DMTP and DNMTP WM in general, or between operant and spatial alternation-based maze assays, specifically. As we had previously failed to train mice in a DNMTP version of the 5-CSWM task^39^, we cannot distinguish between these possibilities. However, a fundamental difference between the role of prefrontal delay-activity in DMTP *vis-à-vis* DNMTP WM is rather unlikely, given that it has been observed in both paradigms in primates^11,13–17^. Also, prefrontal delay-activity has been measured in mice^61^ and rats^62^ during alternation assays as well as in a tactile cue-discrimination WM task^63^.

Instead, the T-maze task could be solved by alternative strategies that are independent from prefrontal delay-activity—like spatial novelty-preference^46^ which was intact in our PFC^ΔNR1^ mice. Similarly, when monkeys are allowed mediating strategies, they can perform a WM task even after a prefrontal lesion^64^. Accordingly, optogenetic manipulations of the PFC or its afferents typically decrease T-maze performance only partially^60,61,65^. In the 5-CSWM task, in contrast, neither novelty-preference nor other mediation strategies can be easily used to maintain task performance in the absence of active WM capability^39^. Additionally, multiple brain regions involved in WM may offer some functional redundancy whereby a deficiency in the PFC may be compensated. This substitution might be easier for some tasks (like the T-maze) than others, given that the involvement of different areas depends on the strategy with which a task is solved^66^. Indeed neural correlates of visuospatial WM are situated across the prefrontal, parietal^67,68^, inferotemporal^69^, and sensory^70,71^ cortices in primates. Our results suggest, however, that the PFC may have a crucial role in those WM tasks in which alternative strategies cannot be easily deployed.

### Prefrontal NMDAR-hypofunction in schizophrenia

Maybe the most striking of the presented findings is the high specificity of the induced WM deficit. In contrast to the T-maze, the 5-CSWM has the advantage that it can simultaneously measure *WM* and the cue-directed *attention* it requires by recording the choice accuracy in the CP and SP, respectively. However, neither the SP accuracy on the 5-CSWM nor the attentional parameters of the subsequent 5-CSRTT were impaired by our prefrontal NMDAR knockout, which replicates a previous report^41^. Also, WM accuracy in PFC^ΔNR1^ mice was neither correlated to nor statistically mediated by such attentional parameters. This suggests that attention and WM—two cognitive functions that are intricately linked to support performance in WM assays—can be dissociated by specific NMDAR-hypofunction in prefrontal excitatory cells.

Two previous studies have established that ablation of NMDARs in prefrontal excitatory cells may cause a specific deficit in cue-discrimination and extinction learning during fear-conditioning, while basic associative learning, sociability, attention, perseveration, and motor impulsivity are intact; social memory was even enhanced^41,42^. Our data broadly support those findings and extend them by demonstrating that the same manipulation also does not affect spatial short-term habituation, rewarded alternation, exploratory or motivational drive, anxiety, or anhedonia. A caveat of these conclusions, however, is the possibility of an incomplete ablation. Analysing PFC^ΔNR1^ mice perfused at different time points corresponding to the beginning and the end of the test battery, we found a consistently low number of Cre-transfected neurons that still displayed GluN1-signal (< 10%), while neurons just transfected with GFP showed a GluN1 signal in ≥ 50% of cells. Nevertheless, this analysis demonstrated that GluN1-ablation in Cre-transfected cells was not complete, and additionally some excitatory PFC neurons were likely not transfected at all. Therefore, we cannot exclude the possibility that such remaining GluN1-positive excitatory neurons might have been sufficient to support normal performance on other tasks—including the T-maze—even if they were ultimately dependent on NMDARs in prefrontal excitatory cells. As such our approach only models moderate GluN1-hypofunction, but not complete GluN1-ablation.

In summary, our data demonstrates that NMDAR-hypofunction in excitatory cells of the prefrontal cortex—a region where NMDAR-expression is indeed reduced in schizophrenia patients^72,73^—may be *sufficient* to cause some of the WM impairments seen in this disease.

## Materials and methods

### Animals

All experiments were performed in accordance with the German Animal Rights Law (Tierschutzgesetz) 2013, the European Union regulations for the use of laboratory animals (EU Directive 2010/63) and the ARRIVE guidelines, and were approved by the Federal Ethical Review Committee (Regierungspräsidium Tübingen) of Baden-Württemberg, Germany (license number TV1285 for the main study, licence number 1399 for the cohort contributing data to Supplementary Fig. 3). 41 male B6.129-*Grin1*^tm1Rsp^/Kctt mice with a homozygous knock-in of a floxed version of the GluN1 (NR1) encoding gene *Grin1* into the native *Grin1* locus (obtainable from EMMA, stock# 09,319) were used for the main study (fGrin1 cohort)^40^. They were 2–3 month old at the beginning of the procedure battery, i.e. at the date of surgery. They were housed in individually-ventilated cages (IVC) containing sawdust, sizzle nest and one cardboard ‘house’ (Datesand, UK) as enrichment, throughout. Temperature (ca. 22 °C), humidity (45–65%) and illumination within a 13 h light / 11 h dark cycle were tightly maintained within the animal holding room. Where possible, mice were kept in groups of 2—5 mice, except during the final experiments (sucrose preference and nest building) lasting 5 days, during which they were deliberately housed in isolation. The mice had ad libitum access to water throughout, and to food prior to and in the interludes between experiments that required appetitively motivated learning. The fGrin1 mice were genotyped from ear-notch samples using primers 5 ‘–TGT GTC CCT GTC CAT ACT CAA–3 ‘ and 5 ‘–AAC ACT GTG GAC CAG GAC TTG–3 ‘ which produce a 325 bp product for the *Grin1*-wildtype locus and a 375 bp product for the floxed *Grin1* allele. During training, they were kept on food-restriction that maintained their weights at no less than 85% of their average individual weights measured during three days prior to the beginning of the food-restriction under ad libitum food access. Except for sucrose preference and nest building which ran overnight, all other experiments, were conducted during the light activity phase which lasted from 7.00 am to 8.00 pm. For analysis of side bias, data from a separate cohort and project was used. This cohort included 11 wildtype littermate controls (10 males) of the *Gria1* knockout (*Gria1*^*–/–*^, B6.129-*Gria1*^tm1Rsp^; MGI:2,178,057; C57BL/6 J background)^74^ line derived from breeding of heterozygous *Gria1*^+*/–*^ parents, kept under the same conditions as stated above and trained in the 5-CSWM task in custom-designed operant boxes^75^.

### Stereotaxic surgery (fGrin1 cohort)

Animals were anaesthetized with isoflurane (5% for induction, 1.5–1.0% for later maintenance; AbbVie, G), their scalps were shaved and disinfected, s.c. injections of analgesics (0.08 mg/kg buprenorphine, Bayer, G; 1 mg/kg meloxicam, Boehringer Ingelheim, G) were applied under the back skin and a local anaesthetic (200 $\upmu$l of 0.025% bupivacaine, AstraZeneca, UK) was injected under the scalp. Animals were placed into either one of two stereotaxic frames (motorized and atlas-integrated frame, Neurostar, G and Kopf, US; manual digital frame, World Precision Instruments (WPI), US) with non-rupture mouse ear bars. A lubricating cream (Bepanthen, Bayer, G) was applied to the eyes to prevent them from drying out and excess illumination during the surgery. A thermometer coupled to a heating pad (Hugo-Sachs, G) underneath the mouse was used to maintain its body temperature at ca. 36–37 °C throughout surgery. Two craniotomies per hemisphere were made above the virus injection sites and suspensions containing either rAAV8-CamKIIα-Cre-GFP (PFC^ΔNR1^ group; titre of 4.3 × 10^12^ vg/ml; University of North Carolina vector core; UNC, US) or AAV5-CamKIIα-GFP (Ctrl group; titre of 4.0 × 10^12^ vg/ml; UNC) were infused at a rate of 100 nl/min via a WPI Nanofil syringe (34 g bevelled needle) using a digital injector pump (Micro4, WPI Instruments). Injection coordinates relative to bregma and injection volumes were for the anterior site: AP 1.7 mm, ML ± 0.25 mm, DV 1.3 mm; 100 nl per site; and for the posterior site: AP 0.6 mm, ML ± 0.3 mm, DV 1.8 and 1.1 mm with infusion volumes of 300 and 500 nl, respectively. In a subset of animals, the PrL was targeted more narrowly using the coordinates for the anterior site: AP 2.5 mm, ML ± 0.3 mm, DV 2.0 mm, 70 nl; and for the posterior site: AP 2.0 mm, ML ± 0.3 mm, DV 2.3 mm, 70 nl. However, post-hoc histology revealed that only in one of these mice the expression was exclusively limited to PrL, while the remainder showed a similar expression as the mice from the former group, involving ACC and partly IL, so that both groups (13 mice with narrow targeting and 7 with broader targeting) were merged as PFC^ΔNR1^ group for analysis.

To avoid backflow of the virus suspension, after each injection the needle was left in place for 5 min, slowly moved up by 0.1 mm and left there for another 5 min before removing the needle out of the brain. Once injections were completed, the scalp was sutured, disinfected and treated with lidocaine/prilocaine cream (ANESDERM Pierre Fabre Dermatologie, G). The mouse was then placed in a warming chamber until it was mobile again, and subjected to post-operative monitoring and care for 7 d, which included s.c. applications of 1 mg/kg meloxicam (Boehringer Ingelheim) on the first 3 d post-surgery.

## Behavioural tests

All behavioural experiments were conducted blind to group identity.

### Operant delayed matching to position (DMTP) 5-CSWM paradigm

This experiment was conducted as previously described^39^. In brief, the 5-choice based operant testing of spatial working memory (5-CSWM) in fGrin1 mice was conducted in commercial operant chambers (ENV-115C-A; Med Associates, VT, US) fitted with an ENV-307A-CT 5-choice wall on one side and a liquid reward receptacle (ENV-303RMA) on the opposite wall fitted with a receptacle light (ENV-302RL-1) and a head entry detector (ENV-303HDA). The chambers were placed individually in melamine-MDF sound-attenuating and ventilated cubicles (ENV-022MD, Med Associates). Strawberry milk (Müllermilch, G) was used as reward and dispensed to the reward receptacle by syringe pumps (PHM-100A, Med Associates) placed outside the MDF-cubicle. All operant behavioural paradigms were implemented by custom-written scripts executed through the Med-PC control software and a control interface (DIG-716P2, housed in a SG-6510DA cabinet; Med Associates). The additional cohort of 11 wildtype mice (Supplementary Fig. 3) was trained in custom-designed operant boxes, described on a dedicated webpage https://github.com/KaetzelLab/Operant-Box-Design-Files and a separate publication^75^.

All experimental stages of the 5-CSWM and prior habituation training were conducted in a dark chamber, by default. Transient illumination resulted either from the poke holes in the 5-choice wall during cue-presentation, from the receptacle light during reward delivery, or from the house light of the chamber during time-out (“punishment”) periods after erroneous responses or omissions.

Before the beginning of the training and coinciding with the beginning of the food restriction, mice were accustomed to the reward as it was placed in their home cages for consumption (Supplementary Fig. 1B). Subsequently, mice underwent a simple 5-light operant training protocol to learn to poke into any illuminated hole to obtain a reward. For this habituation training, all 5 lights of the 5-choice wall were illuminated simultaneously for an unlimited *stimulus duration* (SD). Once the mouse poked into one of the holes, the 5 lights were turned off, the receptacle light was turned on and a 40 $\upmu$l milk reward was delivered. Once consumed (2 s consumption time), a new trial started immediately as the receptacle light turned off and the 5-choice lights turned on. Once mice achieved at least 30 rewards in two consecutive daily 30 min sessions, the actual 5-CSWM began which was also conducted in 30 min daily sessions (Supplementary Fig. 1B).

The 5-CSWM operant cycle (Supplementary Fig. 1A) began with a *sample phase* (SP) during which the mouse had to poke the one hole of the 5-choice wall that was illuminated for a certain *stimulus duration* (SP-SD). If correctly poked within the SP-SD time, illumination of this hole was turned off and the receptacle light was turned on while a 20 $\upmu$l reward was delivered. A time of 2 s was allowed for consumption and ended by turning off the receptacle light. Immediately afterwards, a *delay phase* of 2 s started and was followed by a *choice phase* (CP) during which—for a maximum *stimulus duration* (CP-SD) of 20 s—the mouse was presented with two illuminated holes at the 5-choice wall, of which one was the hole that had been presented in the prior SP and was the correct choice option in this DMTP paradigm. In case of a correct poke into this hole (*correct response*), the cue lights were turned off and the receptacle light was illuminated with simultaneous delivery of a 60 $\upmu$l reward (Supplementary Fig. 1A). All other responses were not rewarded but were followed by a 5 s time-out period during which the house light was turned on. These erroneous responses included pokes into non-illuminated holes during the SP (*incorrect SP response*) or CP (*incorrect*_*unlit*_* CP response*), incorrect responses into the illuminated hole during the CP (*incorrect*_*lit*_* CP response*), or omissions of any response during the allowed SD response times in either phase (*SP omission, CP omission*). Premature pokes had no consequence. An inter-trial interval (ITI) of 20 s (including the 5 s time-out after erroneous responses) followed before a new SP with cue-presentation was started.

The primary readouts during this operant cycle were measures of WM performance, especially *accuracy*_*lit*_ (number of correct CP responses divided by the number of correct and incorrect_lit_ CP responses), but—as surrogate measure—also *accuracy*_*all*_ (number of correct CP responses divided by the number of all CP responses, excluding omissions). We further recorded the absolute *number of correct responses* and *%omissions* (number of CP omissions divided by the number of conducted CPs (i.e. correct SP responses)) as indicators of task engagement. In the SP, levels of attention paid towards the to-be-remembered stimulus were determined primarily from the SP *accuracy* (number of correct SP responses divided by the number of all SP responses, excluding omissions), with surrogate measures *number of correct responses*, and *%omissions* (number of SP omissions divided by the number of conducted SPs (i.e. trials)—analogous to the readouts of the 5-CSRTT^43^. Furthermore, the time that the animal took from the exit from a correct poke to the entry into the reward receptacle in the CP was defined as *reward latency* and used as a compound measure for locomotor drive and motivation, as usually done in the 5-CSRTT (Supplementary Fig. 1A). Additional control variables included the number of *correct* and *incorrect premature responses* made during the delay and CP, and the *true total delay*, which represents the time between the poking of the correct hole in the SP and poking in the CP (sum of CP-initiation latency, which can be regarded as SP reward latency, the set delay in a given protocol and the CP response latency. Premature responding was only recorded in a subset of animals and may represent a mediation strategy whereby an animal refreshes its memory during the delay phase by poking into what it regards as the correct hole^39^.

To aid task acquisition, the 5-CSWM was trained in increasingly difficult stages through which the mice transitioned depending on achieving certain performance criteria during 3 consecutive days on the prior stage. The parameters and performance criteria of these stages are stated in Supplementary Fig. 1B) and are described in detail in our prior publication of this task^39^. Essentially, in the first three stages the delivered SP reward was reduced from 20 to 10 to 0 $\upmu$l to focus the animal’s performance goal on success in the *CP*, while the number of possible choice configurations in the CP was increased. In stage 4, additionally the SP-SD was reduced from 20 to 8 s. Stage 4 was also the baseline protocol used for further training and the baseline test for all challenge protocols. Delay challenges varied from the baseline by applying a longer delay (12 s or 22 s instead of 2 s) after the receptacle poke. For the distraction challenge, a delay of 5 s (instead of 2 s) was used during which two pseudo-randomly timed switches of the house-light (two times 0.5 s) were applied. Mice transitioned to the next stage, if—in three consecutive daily 30 min sessions—the accuracy_lit_ was ≥ 70% and the number of correct choices was ≥ 10, or if they had done a maximum number of sessions in a stage (58 sessions at stage 1, 17 sessions at stage 2 and 17 sessions at stage 3, 19 sessions at stage 4). Mice needed to be trained for up to 21 weeks to be tested in the challenges.

### 5-choice serial reaction time task (5-CSRTT)

Having initially learnt the OP WM task and performed the challenges, the mice started the training phase on a 5-CSRTT protocol. This task corresponded to the SP of the 5-CSWM albeit with a waiting time (ITI) of 5 s before cue-presentation and an SD that was increasingly shortened from 8 to 4 s (trained for 16–19 sessions) and to 2 s (baseline stage before challenges started)^76^. Also, the default-state of the house-light was to be switched *on* in this task, while time-outs for erroneous responses involved to switch the house-light off. Correct responses were rewarded with a 20 $\upmu$l milk reward. An additional 2 s to respond to the cue was allowed after the end of the SD (limited-hold time). Furthermore, premature pokes into any hole during the ITI (i.e. before the SD) were recorded and also punished by immediate cancellation of the trial, including omission of the reward and initiation of a 5 s time-out. The number of premature responses relative to the total amount of trials (%prematures) were taken as the primary indicator for motor impulsivity assessed in this task^43,76^. On the baseline stage, the ITI was 5 s, and the SD was 2 s. For testing of attention and impulse control, these two functions were challenged individually in separate protocols involving either the reduction of the SD to 1 s (attention challenge) or the elongation of the ITI to 9 s (impulsivity challenge).

### T-maze: delayed non-matching to position (DNMTP) paradigm

The delayed non-matching to position (DNMTP) paradigm of spatial WM was investigated using rewarded alternation on the T-maze. The T-shaped structure (W 10 cm, L 40 cm; H 10 cm) consisted of a red PVC floor and transparent Perspex walls with white plastic food wells at the end of each goal arm. During the experiment the mice were kept under food restriction that maintained their weights at 85–90% of their free-feeding weight. The reward used for this experiment was a 2:1 dilution of condensed milk (‘Ja’, REWE, G) in their drinking water. First, the mice were habituated to the maze in groups (with cagemates) and then individually to reduce anxiety before training and testing, and to ensure reward consumption. For both training and testing, 10 trials per session (day) were performed and each trial consisted of a sample (SP), a delay (DP) and a choice phase (CP). In the SP, mice were placed in the start arm and allowed to enter a randomly determined goal arm to consume the reward, while the alternate arm was blocked with a non-transparent barrier. The identity of the SP goal arm was determined pseudo-randomly, whereby no more than three consecutive trials could have the same goal arm and an equal allocation of right and left goal arms was used for each session. After gaining the reward in the SP, the mouse was removed from the maze for a 5 s DP, while the barrier blocking the previously inaccessible (novel) goal arm was removed, while all other spatial and odour cues present in the SP were maintained. Subsequently, the mouse was returned to the start arm for the CP and left to choose between the novel, rewarded and the familiar, unrewarded arm. 10 training sessions were conducted with a 5 s delay and 5–7 min inter-trial-interval (ITI) using a round robin arrangement. After those 10 training sessions, the mice were subjected to challenges during which the delay time between the SP and the CP was altered (1 s, 20 s and 60 s), with one specific delay applied for two consecutive days. In the 1 s challenge, a massed design was used where all 10 trials were performed consecutively by each mouse, with an ITI of 20–25 s between them^45,76,77^. For the other two challenge protocols, a blocked design was used with an ITI of at least 3 min. The number of correct choices divided by the number of trials per session was calculated as a readout of WM performance (WM accuracy), and for the challenge protocols the accuracy was averaged across the two days with equal delay.

### Y-maze spatial novelty preference test

Spatial short-term habituation was tested as spatial novelty-preference in a Y-shaped maze with three transparent arms (20 cm high, 30 cm long, 8 cm wide) uniformly separated by 120°. The floor of the maze was covered with clean sawdust that was interspersed with some dirty sawdust collected from the home-cage of an unfamiliar same-sex group of mice. Before the test, each mouse was kept in a holding cage near the Y-maze for 8–10 min. The test began with a 5 min SP where the mouse could explore the start arm and one goal arm (counterbalanced between groups). The alternate goal arm was blocked by a non-transparent grey PVC barrier. At the end of the SP, the mouse was transferred back into the holding cage for a 1 min DP during which the barrier to the previously inaccessible arm was removed and the saw dust from all arms was mixed and dispersed within the maze. The mouse was subsequently returned to the maze for a 2 min test phase which allowed access to all arms. ANY-maze (San Diego Instruments, US) was used to track the movement of the mouse during the experiment. Novelty preference was analysed by calculating the *time* spent in the novel goal arm divided by the sum of the time spent in both goal arms. Additionally, a preference score was calculated based on the *number of entries* into the novel goal arm divided by the sum of the number of entries into both goal arms.

### Novelty-induced locomotor activity measurement (LMA)

Locomotor activity was tested in a novel open field (OF), which was a clear plastic cage (425 × 266 × 185 mm; Eurostandard Typ III, Tecniplast, G) filled with clean sawdust. Movements were recorded for 90 min using CCTV cameras (Sentient, UK) installed centrally above the open-field cages. The video-recordings were fed into a single image frame through a CCTV-system (Dahua Inc, China), digitized through an A/D converter (TheImagingSource, G), and processed by ANY-maze (San Diego Instruments, US) to extract the distance moved in 5 min time intervals as well as the average distance to the OF border.

### Sucrose preference test (anhedonia)

Testing was conducted over four consecutive nights, i.e. during the dark phase of the day. In each night, single-housed mice had access to two bottles of water placed on the same side of the cage. On the first night, mice were habituated to being single-housed and to the presence of the two bottles filled with water. Over the following nights one of the bottles was consistently filled with plain water, while the other bottle was filled with sucrose-containing water, whereby the sucrose concentration was either 1% (nights 2 and 3) or 10% (night 4). Every morning and evening, the bottles were weighed to measure consumption, and those bottles containing sucrose solution were removed from the cages, leaving the mice with access to plain water during the day. The preference for sucrose in each night was calculated as a percentage of sucrose solution consumption over total liquid intake.

### Nest building

The nest building experiment was conducted in the night after the last sucrose-preference test. The mice were single-housed in cages with saw-dust covering the floor. All previous enrichment of the cage was removed. A single 2.3–2.6 g Nestlet (Datesand, UK) was introduced to each cage and left overnight to the mice to build nests from the material^50^. The resulting nest and leftover Nestlet material were evaluated on the next morning. The quality of the nest was rated by two observers according to an established scoring scale^50^ and the unused Nestlet material was weighed.

### Reciprocal social interaction (sociability)

We investigated reciprocal social interaction in the same type of cages as used for the LMA test, whose floor was covered with fresh sawdust. A young adult, male stimulus mouse was inserted into the cage for 2–5 min before the test. Each fGrin1 mouse was put in the cage and left to interact with the stimulus mouse for 16 min, while their behaviour was recorded using a CCTV camera (Sentient) placed above the cage and a CCTV recorder (Dahua). Social interactions such as sniffing and grooming, displayed by the fGrin1 mice were scored blind to genotype from the video files in 2 min intervals, while aggression or sexual behaviour was not counted as social interaction. One (PFC^ΔNR1^) mouse was excluded from the analysis due to repeated aggressive behaviour, which was otherwise not observed in our cohort during this test.

### Elevated plus-maze (anxiety)

The elevated plus maze (EPM) was used to test for unconditioned anxiety. The cross-shaped maze was custom-made from grey PVC and consisted of four arms with identical area (L 35.5 cm, W 7 cm), whereby two opposing arms were open, while the other two were enclosed by walls of 20 cm height. The arms were connected by a neutral centre (L 7 cm, W 7 cm). The maze was elevated 70 cm above the ground and the area underneath the maze was covered with cloth to protect the mice in the eventuality of a fall during the test. Light intensity in the maze was 100 lx on open arms and 75 lx in the centre. A test mouse was at first placed in a novel holding cage in the testing room for a 5–7 min habituation period. Thereafter, the mouse was transferred to the centre of the maze and allowed to explore the maze for 5 min. The movement was tracked and analyzed using ANY-maze. Entries into each zone were defined according to the position of the mouse’s body centre. A preference score was calculated as the time spent in the open arms divided by the time spent in the closed arms (excluding the centre). This measure represents the primary, inverse measure of unconditioned anxiety in relation to exploratory drive.

## Histology

### Histological validation of GFP expression

Upon completion of the behavioural tests, mice were transcardially perfused under deep, terminal anaesthesia induced by a mixture of ketamine and medetomidine (≥ 200 mg/kg ketamine, Zoetis, G; ≥ 2 mg/kg medetomidine, Pfizer, US) injected i.p.. After perfusion with phosphate-buffered saline (PBS, Sigma, D) and, thereafter, 4% paraformaldehyde (PFA) in PBS (SantaCruz, US), the brain was post-fixed in 4% PFA/PBS for one night, followed by incubation in a storing solution (0.05% sodium azide in PBS) at 4 °C. Then, the PFC of the fixed brain was incubated for at least 1 h in a cutting solution (0.05% sodium azide and 10% sucrose in PBS) and cut into 60 µm coronal slices using a vibratome (VT1000, Leica, G) at room temperature (RT). Subsequently, the slices were transferred onto a multi-well plate filled with storing solution at 4 °C. Slices were washed with PBS (3 × 10 min), stained with DAPI (0.0005% w/v DAPI in PBS) for 20 min, washed again in PBS (3 × 10 min), and mounted on glass slides in VectaShield (H-1000; Vector Labs, US) to assess expression of GFP(-Cre) with an epifluorescence microscope (DM6, Leica).

### Immunohistochemistry (IHC)

To evaluate the ablation of GluN1, tissue was used from 13 mice transfected with either the GFP vector (6 mice, of which one did not express) or GFP-Cre vector (7 mice) also used for the behavioural cohort (see details above), and perfused after 3–4 or 7 weeks, as described above. Brains were processed as described above, and 40 µm thick coronal brain slices from the PFC were cut and transferred into CELLSTAR (Cat# 662,160 Greiner Bio-One, Austria) multi-well cell-culture plates. Additionally, 60 µm thick slices from 8 Cre-transfected brains from the behavioural cohort were used for IHC analysis. Slices were washed in citrate buffer (Antigen Retrieval Citra Plus Solution, HK086-9 K, BioGenex, US) for 10 min at RT. Using a microwave, the slices were then boiled in citrate buffer. After cooling, the slices were washed twice in PBS for 10 min at RT, then immersed in a detergent (0.15% TritonX/PBS) for 10 min. In order to block unspecific binding sites, the slices were kept in blocking solution containing 0.15% TritonX, 10% normal horse serum in PBS for 1 h at RT.

The slices were then incubated over two nights at 4 °C in carrier solution containing the primary antibody and 0.05% TritonX, 2% normal horse serum in PBS. The following primary antibodies were used: polyclonal rabbit anti-GluN1 (order# AGC-001, Alomone labs, Israel; diluted 1:300), monoclonal mouse anti-Gad67 (order# MAB5406, Merck/Millipore, G; 1:500), and polyclonal chicken anti-GFP (order# ab13970, Abcam, UK; 1:500). Immuno-labelling of GFP was only conducted in slices from the behavioural cohort as they were processed several months after perfusion; in the other 14 mice native GFP-expression was used as a counter-label. After this incubation, slices were washed twice for 10 min in PBS at RT, and subsequently stained for 2 h in light-safe conditions at RT with a secondary AlexaFluor-546-labelled anti-rabbit antibody (order# A-11010, Invitrogen/ThermoFisher, US) diluted at 1:1000 in carrier solution containing 0.05% TritonX, 2% normal horse serum in PBS. Slices were then washed twice for 10 min in PBS at RT, incubated for 10 min in a DAPI-solution diluted in PBS, and washed twice for 10 min in PBS at RT. After the slices were mounted on microscopy slides, they were embedded in VectaShield antifade mounting medium (VectorLabs) and stored at 4 °C until epifluorescence imaging (DM6, Leica).

### Statistical analyses

Statistical analyses were performed using SPSS. Two-way repeated-measures analyses of variance (2-way RM ANOVA) were used in experiments that included multiple challenge protocols, training data, or a baseline measure before a challenge. Significant effects in this ANOVA were further investigated using simple main-effects pairwise post-hoc tests with Sidak adjustments for multiple comparisons. Inclusion of covariates in the 2-way RM-ANOVA and bivariate Pearson correlation analysis were used to assess dependencies of WM accuracy on other task parameters. *T*-tests were used on normally distributed data with a simple between-subjects design. Its non-parametric equivalent, the Mann–Whitney-U (MWU) test was used where the normality of the data was not assumed. A *P* < 0.05 was considered statistically significant. All data are presented as mean values ± standard error (s.e.m.) or as mean values ± standard deviation (S.D.), as indicated, or as dot plots representing the values of each individual animal.

## Data availability statement

All data generated in this study as well as Med-PC scripts of the operant tasks can be obtained upon reasonable request from the corresponding author.

## Supplementary Information

Supplementary Information
