# Supplementary material for: Delayed-matching-to-position working memory in mice relies on NMDA-receptors in prefrontal pyramidal cells
Source: Sci Rep. 2021 Apr 22;11:8788. doi: 10.1038/s41598-021-88200-z (PMC8062680; doi:10.1038/s41598-021-88200-z)
Supplement: Supplementary file 1 — Supplementary Information [file 41598_2021_88200_MOESM1_ESM.docx]

Supplementary Information

# Delayed-matching-to-position working memory in mice relies on NMDA-receptors in prefrontal pyramidal cells

**Authors:**

Kasyoka Kilonzo^1^, Bastiaan van der Veen^1^, Jasper Teutsch^1,2^, Stefanie Schulz^1^, Sampath K.T. Kapanaiah^1^, Birgit Liss^1,3^, Dennis Kätzel^1,*^

^1^ Institute of Applied Physiology, Ulm University, Ulm, Germany

^2^ *Present address:* Newcastle University, Newcastle upon Tyne, UK

^3^ Linacre College and New College, University of Oxford, Oxford, UK

* Correspondence: dennis.kaetzel@uni-ulm.de; +49 731 500 33770; Fax +49 731 500 33779; Institute of Applied Physiology, Ulm University, Albert-Einstein-Allee 11, 89081 Ulm, Germany

# Supplementary Figures


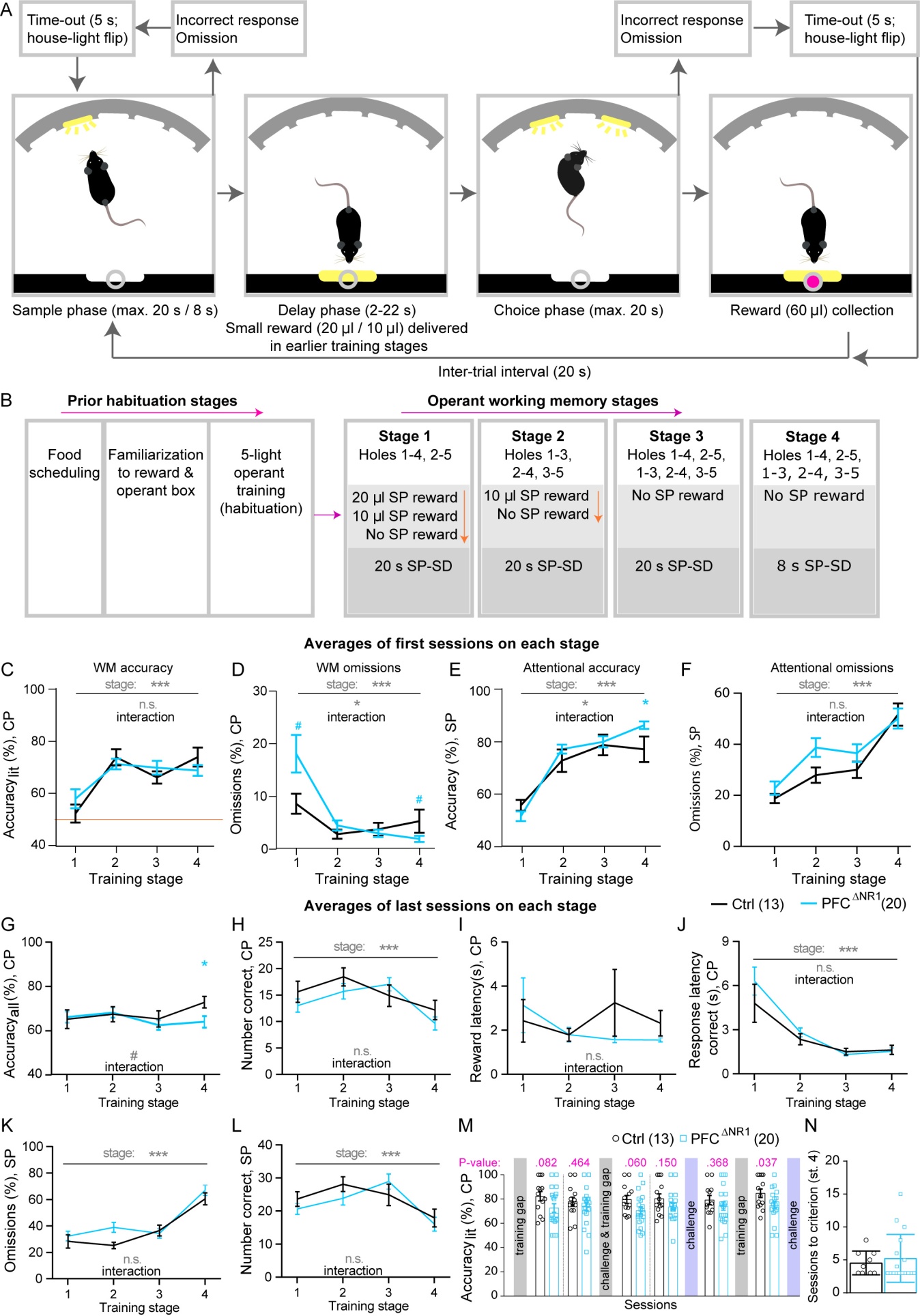


**Supplementary Figure 1. Operant cycle, training stages and training performance in the 5-CSWM task.** (**A**) 5-choice based operant testing of spatial working memory (5-CSWM): mice need to poke into an illuminated hole in the sample phase (SP), return to the opposite wall during the delay phase, and then poke the previously illuminated hole in the choice phase (CP) to obtain a large reward (pink). Omissions or pokes into incorrect holes during the SP or CP are punished by a cessation of the ongoing trial, the resulting lack of reward, and a 5 s timeout during which the house-light is switched on (as animals are otherwise trained without illuminated house-light). (**B**) Training schedule for acquisition of the task, with coloured arrows representing transitions from one stage to the next over time, depending on performance (see Methods). Prior habituation stages (left) include food restriction, delivery of the milk reward in the operant box and the acquisition of the basic operant cycle in which mice need to poke any hole of the 5-choice wall (all of which are illuminated) to obtain a reward. Subsequently, the 5-CSWM training starts (right), whereby the number of options of co-illuminated holes in the CP increases across the main stages (pink arrow), and the amount of milk reward (orange arrow) decreases or, later, the stimulus duration (SD) in the SP decreases. (**C-F**) Performance of mice trained in the 5-CSWM task over 4 stages, averaged within the *first* 3 training sessions on each stage; shown are the primary indicator of WM performance CP *accuracy_lit_* (**C**), indicators of task engagement, *% omissions* in the CP (**D**) and SP (**F**), and the primary attention indicator *accuracy* measured in the SP (**E**). (**G-L**) Further indicators of performance of mice trained in the 5-CSWM task over 4 stages, averaged within the *last* 3 training sessions on each stage (as analysed in Fig. 2B-D); shown are the secondary WM indicator *accuracy_all_,* (**G**), the total *number of correct responses* in the CP (**H**) and SP (**L**), the CP reward (**I**) and response (**J**) latencies, and the SP %omissions (**K**). (**M**) WM *accuracy_lit_* for individual days, in chronological order, where mice were trained under baseline (stage 4) conditions before and between the three challenges with training gaps of 1-2 d indicated by grey bars; *P*-values from individual t-tests are stated above the bars in magenta. (**N**) Number of sessions required to reach criterion for the final stage (4) on that stage indicates no difference between genotypes in acquiring the task. Note that 4 Ctrl mice and 3 PFC^ΔNR1^ mice did not reach criterion in the permitted number of sessions before challenge protocols commenced. Grey statistical indicators in (C-L) refer to the indicated factors of the RM-ANOVA across the four stages and two groups, with an overall effect of stage shown above the horizontal line in grey, and interactions below in grey; no significant effects of group were found. The legend in (J) indicates the colour-code and size of each group. See Supplementary Table 2 for statistical details on RM-ANOVAs for all assessed parameters. n.s., *P* > 0.1, ^#^ *P* < 0.1, * *P* < 0.05, *** *P* < 0.001. Error bars, s.e.m. (C-M) or S.D. (N). Panels (A) and (B) have been reproduced from ref. 39.


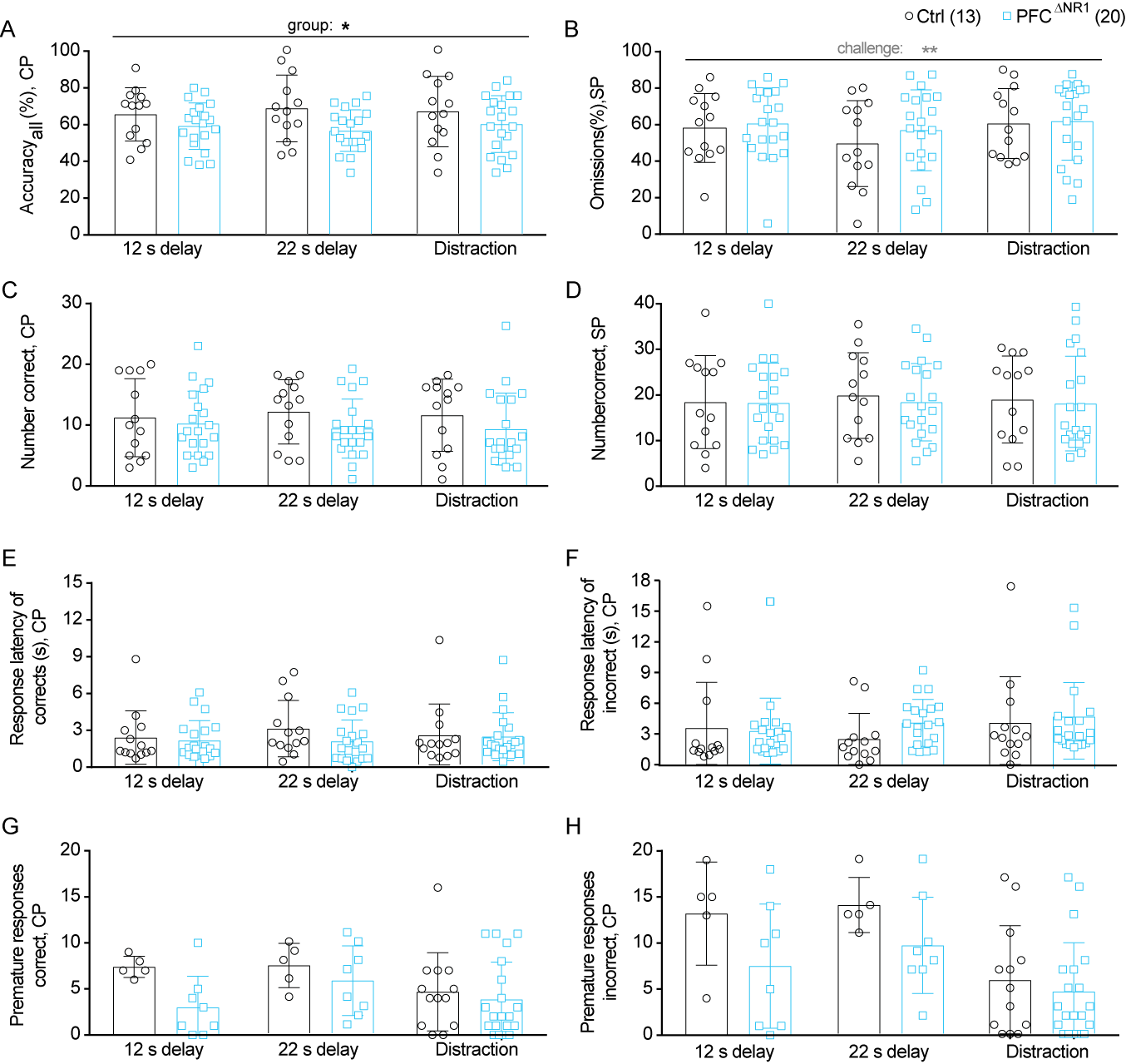


**Supplementary Figure 2. Performance in 5-CSWM task challenges.** (**A-H**) Performance indicators from the 5-CSWM for the challenges for which primary indicators are shown in Fig. 2F-J. Statistical indicators in every data panel relate to RM-ANOVA across challenges and groups, stating effects of challenge (grey) or group (black) where present; no significant group-protocol interactions were found, see Supplementary Table 2 for statistical details on RM-ANOVAs for all assessed parameters. The legend above (B) states the colour-code and size of each group. For the two delay challenges, premature responding was only recorded in a subset of animals, causing a lower *N*. * *P* < 0.05, ** *P* < 0.01. Error bars, S.D.


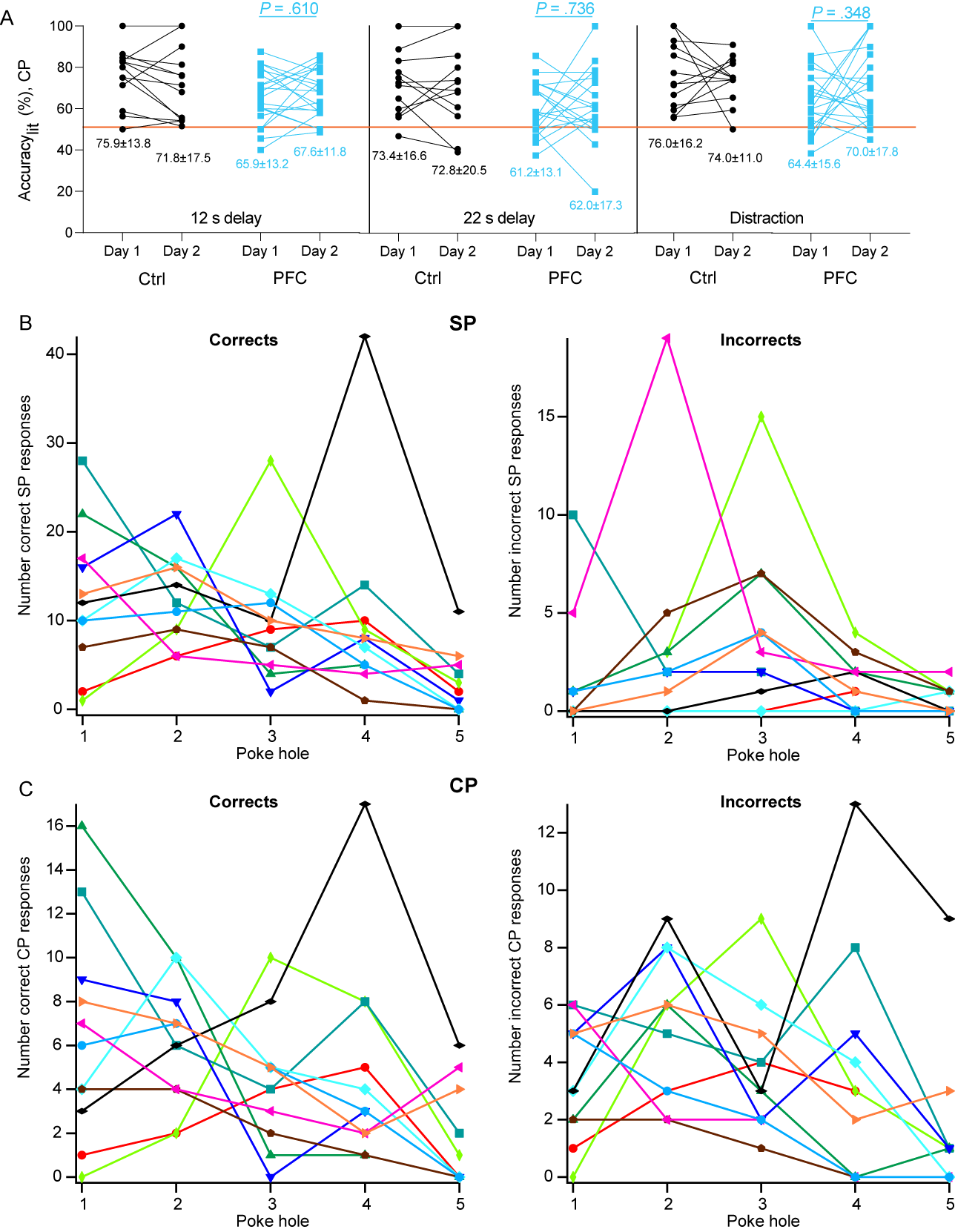


**Supplementary Figure 3. Assessment of individual challenge days and of individual bias for poking into select holes.** (**A**) WM accuracy shown for each group (black, controls; cyan, KO), challenge protocol (named above x-axis), and challenge session (day, stated below x-axis). Performance between sessions has been compared within the KO group with paired t-tests (*P*-values stated above). Group means ± standard deviation are stated below the respective data points they refer to. (**B-C**) Number of correct (left) and incorrect (right) pokes into each of the 5 holes of the 5-choice wall in the sample phase (**B**) or choice phase (**C**) assessed in a separate group of 11 wildtype mice, coded by the same colour in each panel. The mice were trained in the same 5-CSWM task (baseline stage, 2 s delay; data shown for a single session) in custom-made operant boxes which allowed to store the information of individual events and hole-pokes (see *Methods* and also https://github.com/KaetzelLab for operant box design files and task code). Note that mice do not have a strong side-bias which could serve as a mediation strategy to solve the task by omitting responses in one half of the box as a general strategy. Given that holes are presented at equal distribution (20% per hole) in the SP, and correct and incorrect responses are the only choice options aside from omissions, the latter do not have a stronger side-bias than what can be appreciated from correct and incorrect responses.

# Supplementary Tables

**Supplementary Table 1. Inclusion and reasons for exclusion of experimental animals.**


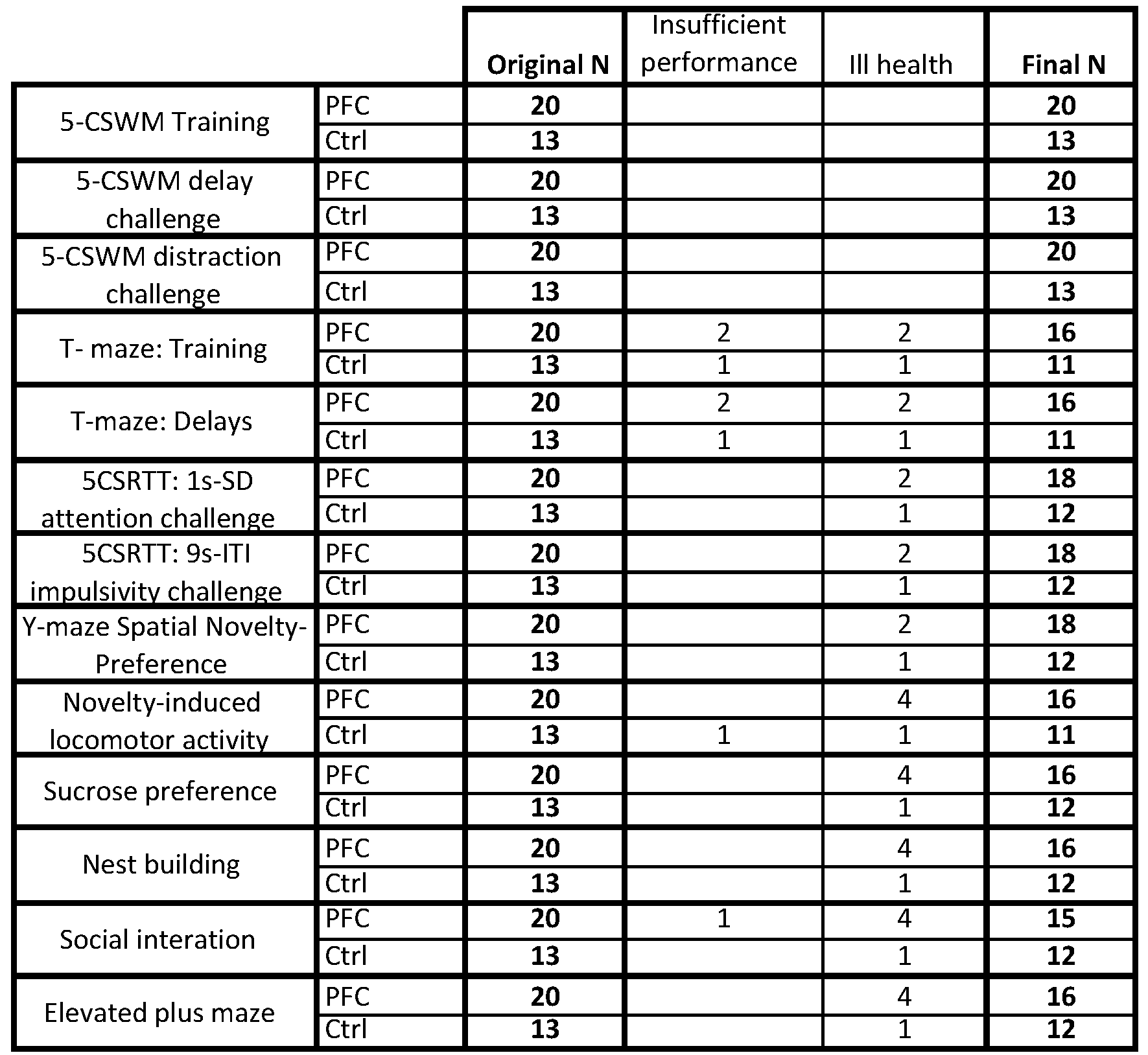


Reasons for exclusion of animals. Animals that were part of the cohorts by histological criteria (stated as “Original N”) but did not contribute data to individual datasets for the stated reasons. The column *Insufficient performance* lists mice that did not participate in the test, because they did not participate in the task, or, in the case of social interaction, displayed repeated aggression. One Ctrl animal was excluded from the LMA dataset as an extreme outlier. The column *Ill health states* animals that were not run in the stated task, but were prematurely perfused because of signs of compromised health (unrelated to the testing procedures).

**Supplementary Table 2. Performance in the 5-CSWM DMTP WM task.**

**
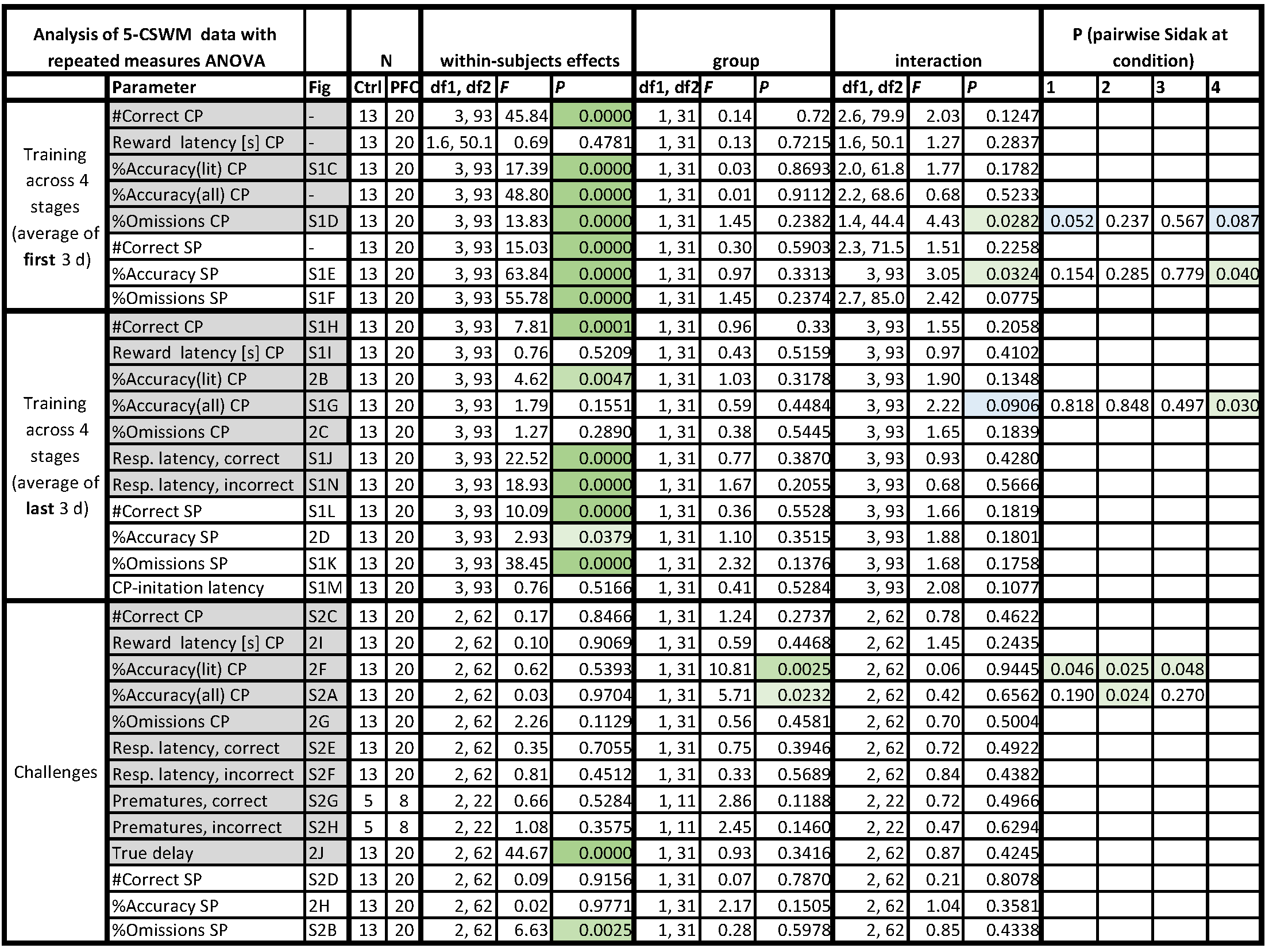
**

Results of repeated-measures (RM) ANOVAs for the experiments shown in Fig. 2 and in Supplementary Figs. 1 and 2. All RM ANOVAs are two-way ANOVAs involving one within-subject parameter (training stage, top and middle; challenge protocol, bottom) and one between-subject parameter (group). The respective experiment or challenge protocol and the statistically tested behavioural parameter are identified in the two left-most columns; the figure that displays the statistically tested data is shown in the figure panel indicated in the “Fig” column. The subsequent columns contain *N*-numbers for each group, and the degrees of freedom (df), *F*- and *P*-values for the within-subject factor, the between-subject factor (group), and their interaction. In case of significant effects of group or interaction, the *P*-values of pairwise (between-subject) post-hoc comparisons at each within-subject condition are stated in the last columns; for the training, conditions 1-4 refer to training stages 1-4; for the challenges, conditions 1-3 refer to the challenges with delays of 12 s (1) and 22 s (2), and for the distraction challenge (3), respectively, analysing the first day of each challenge. Parameters stated on grey background refer to the CP, the others to the SP of the 5-CSWM task. Degrees of freedom and *P*-values have been adjusted according to Greenhouse-Geisser if sphericity could not be assumed. For the two delay challenges, premature responding was only recorded in a subset of animals, causing a lower *N*. *P*-values <0.1 are highlighted in light blue, *P*-values < 0.05 in light green, *P*-values < 0.01 in darker green, and *P*-values < 0.001 in dark green.

**Supplementary Table 3. Assessment of covariation between DMTP WM performance and potentially mediating parameters.**


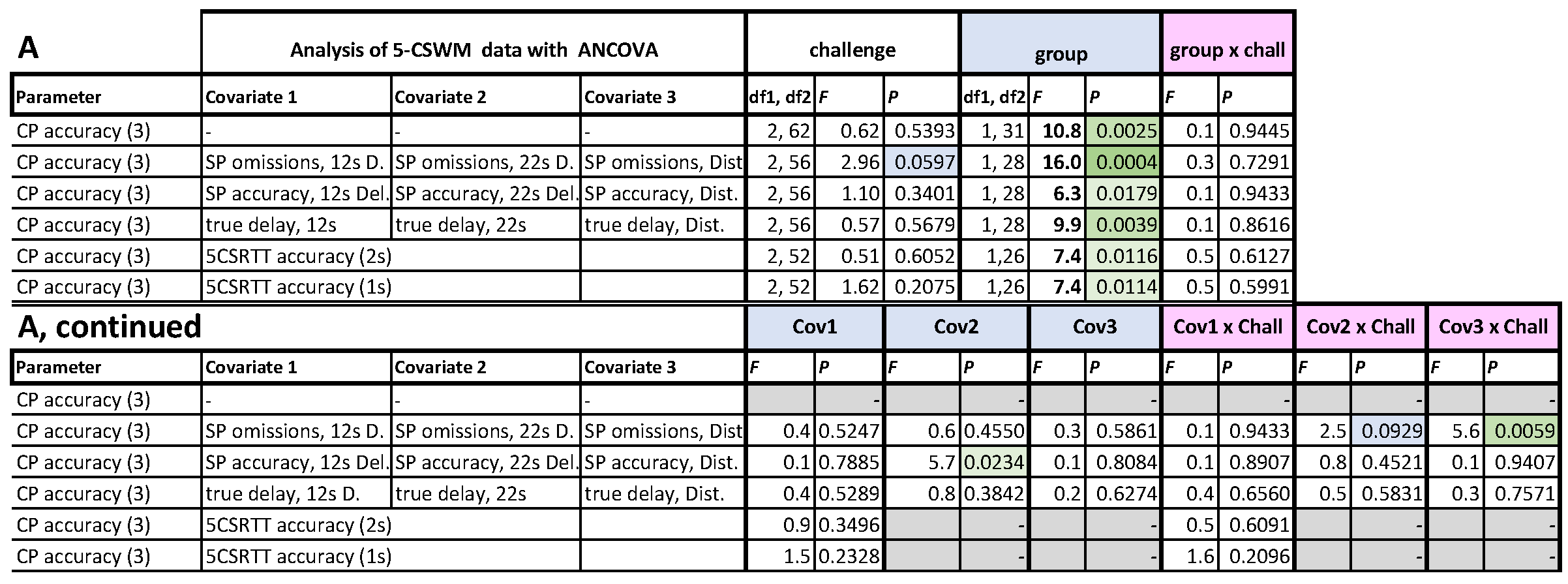


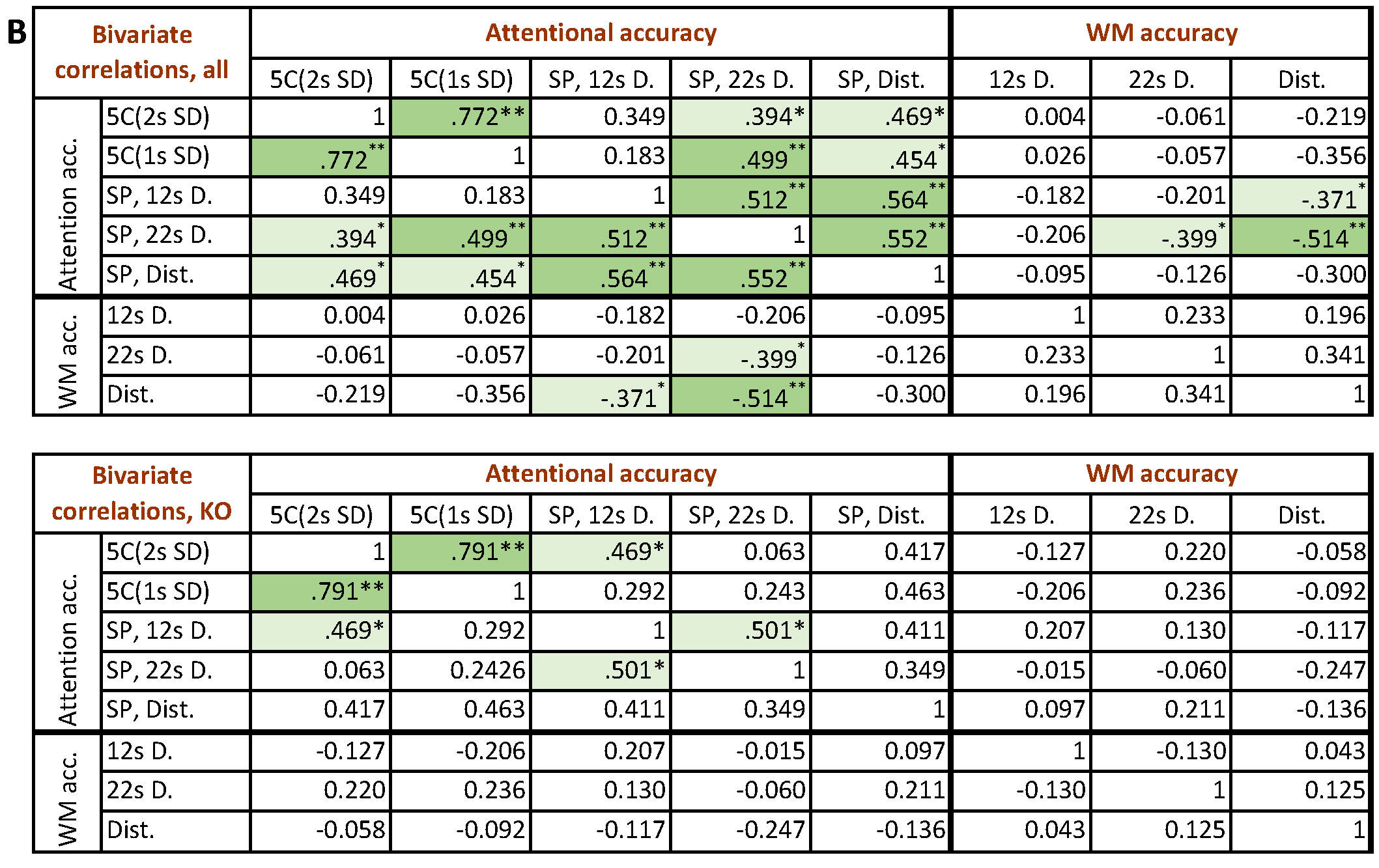


(**A**) Results of repeated-measures ANCOVAs assessing the effect of group on 5-CSWM performance across the three challenge conditions (3) displayed in Fig. 2 (first day of each challenge), but including attentional parameters (accuracy in SP of the three protocols or accuracy in 5-CSRTT, 5C, with an SD of 2 s or 1 s), SP omissions, or the true delay as covariate. *P* < 0.001, dark green; *P* < 0.01 medium green; *P* < 0.05 light green; *P* < 0.1, light blue. Note that the inclusion of attentional accuracy parameters – but not SP omissions - decreases the effect size (F, bold) of *group*. (**B**) Bivariate correlation analysis to further explore the dependencies between WM accuracy and attentional accuracy. Correlated attentional and WM accuracies are the same parameters as those used in (A). Correlations were either conducted across both groups (top) or within the KO-group only (bottom). While attentional parameters correlated strongly with each other in both groups, WM accuracies did not correlate with attentional accuracies within the KO group at all, and only in one case (22 s delay challenge) SP accuracy correlated with CP accuracy within the same challenge when correlating across both groups (all; see main Figure 2K). Additionally, when correlating across all mice, WM accuracy in the distraction challenge correlated with SP accuracy in the other two challenge conditions. Note that, in all three cases, the correlations are *negative*, i.e. higher SP accuracy relates to lower WM accuracy and the reverse. D., delay challenge protocol; Dist., distraction challenge protocol.

**Supplementary Table 4. Assessment of schizophrenia-related behavioural deficits.**


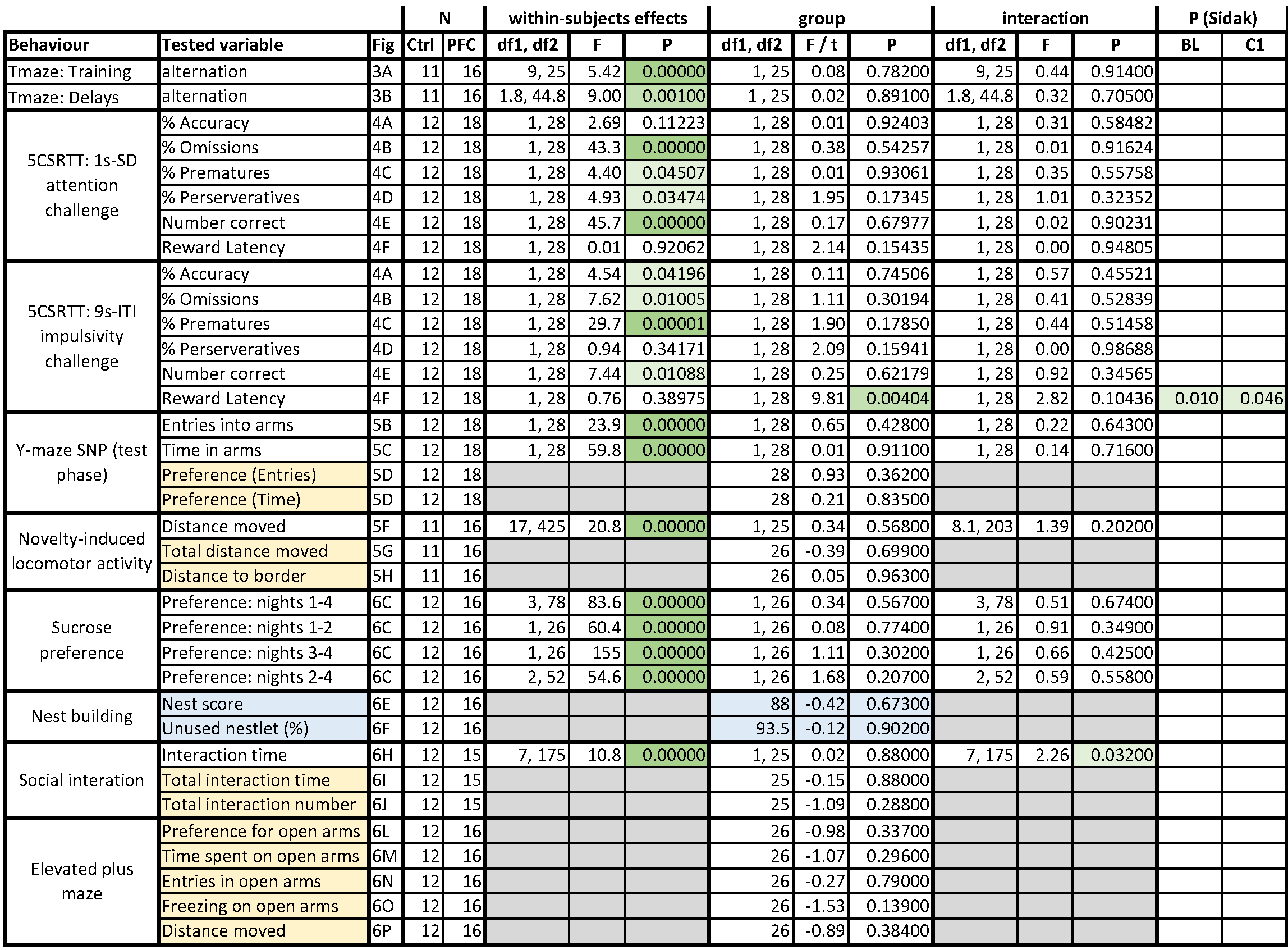


Statistical analysis of the experiments and behavioural parameters shown in Figs. 3-6 and stated in the first two columns. The colour of the second column indicates, if the tested variable was assessed with a Mann-Whitney-U-test (MWU, light blue), a *t*-test across the two groups (yellow), or a two-way RM ANOVA involving one within-subject parameter (as named) and one between-subject parameter (group). The figure that displays the statistically tested data is shown in the figure panel indicated in the “Fig” column. The subsequent columns contain *N*-numbers for each group, and the degrees of freedom (df), *F*- and *P*-values for the within-subject factor (if present, otherwise grey), the between-subject factor (group) and their interaction (if present, otherwise grey). Where a *t*-test has been used, t-value is stated, instead of the F-value, and where the MWU-test has been used the MWU- and Z-values are displayed instead of the df1/2 and F-values. In case of significant effects of group or interactions, the *P*-values of pairwise (between-subject) post-hoc comparisons at each within-subject condition are stated in the last columns; for the 9 s ITI 5-CSRTT challenge the conditions 1-2 refer to baseline (5 s ITI) and 9 s ITI respectively. See Supplementary Table 1 for reasons for exclusions and variations of *N*-numbers across experiments. *P*-values < 0.05 are highlighted in light green, *P*-values < 0.01 in darker green, and *P*-values < 0.001 in dark green.
